# Supplementary material for: AICDA drives epigenetic heterogeneity and accelerates germinal center-derived lymphomagenesis
Source: Nat Commun. 2018 Jan 15;9:222. doi: 10.1038/s41467-017-02595-w (PMC5768781; doi:10.1038/s41467-017-02595-w)
Supplement: Supplementary file 1 — Supplementary Information [file 41467_2017_2595_MOESM1_ESM.pdf]

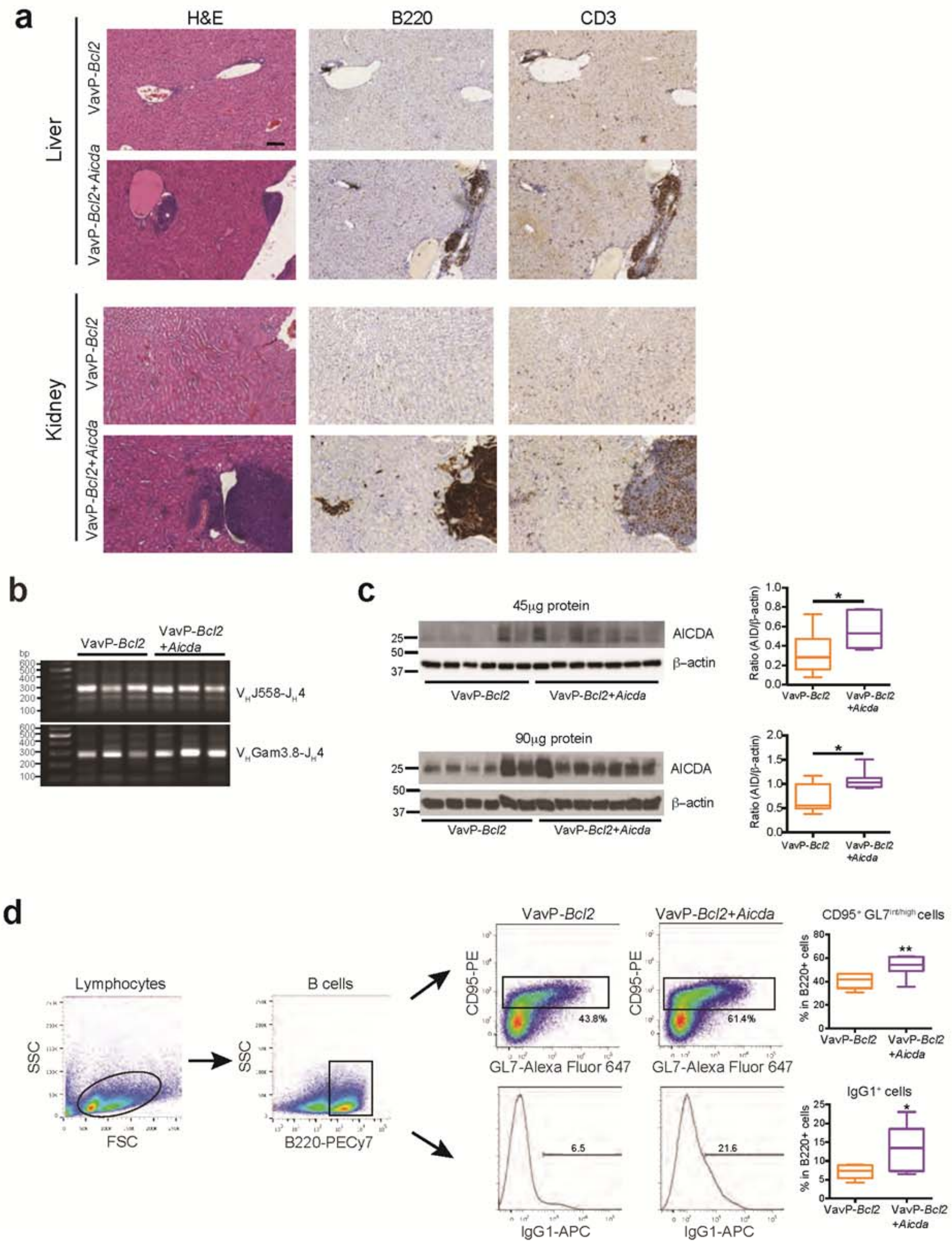

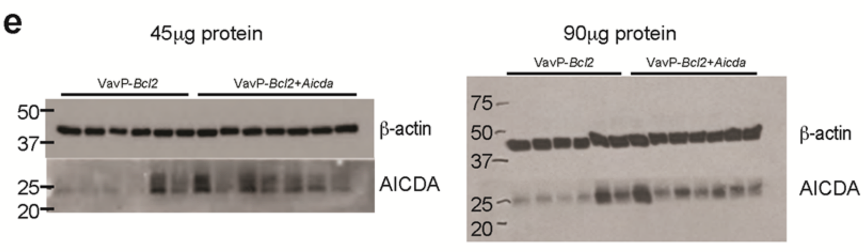

Supplementary Figure1. AICDA overexpression results in more aggressive BCL2-driven lymphomas. (a) Representative histologic sections of formalin-fixed paraffin-embedded liver and kidney tissues from VavP-*Bcl2* and VavP-*Bcl2+Aicda* mice. Sections were stained with H&E and antibodies specific for B220 and CD3. Scale bar represents 100µm. (b) Tumor clonality analysis by PCR of VHJ558-JH4 and VHGam3.8-JH4 heavy chain rearrangements performed in B220+ lymphoma cells from spleens of VavP-*Bcl2* (n=3) and VavP-*Bcl2+Aicda* (n=3) mice. (c) Immunoblot of AICDA and β-actin (left) and AICDA/β-actin expression ratio (right) in splenocytes from VavP-*Bcl2* (n=6) and VavP-*Bcl2+Aicda* (n=7) mice. 45µg and 90µg of loaded protein are shown. (d) Representative flow cytometry plot and quantification of B220+CD95+ and B220+IgG1+ cells in the spleens of VavP-*Bcl2* (n=6) and VavP-*Bcl2+Aicda* (n=7) mice. Box plot represent mean, first and third quartile and whiskers indicate minimum and maximum of all data; two-tailed t test, *P < 0.05, **P < 0.01. (e) Uncropped blots for panel c.

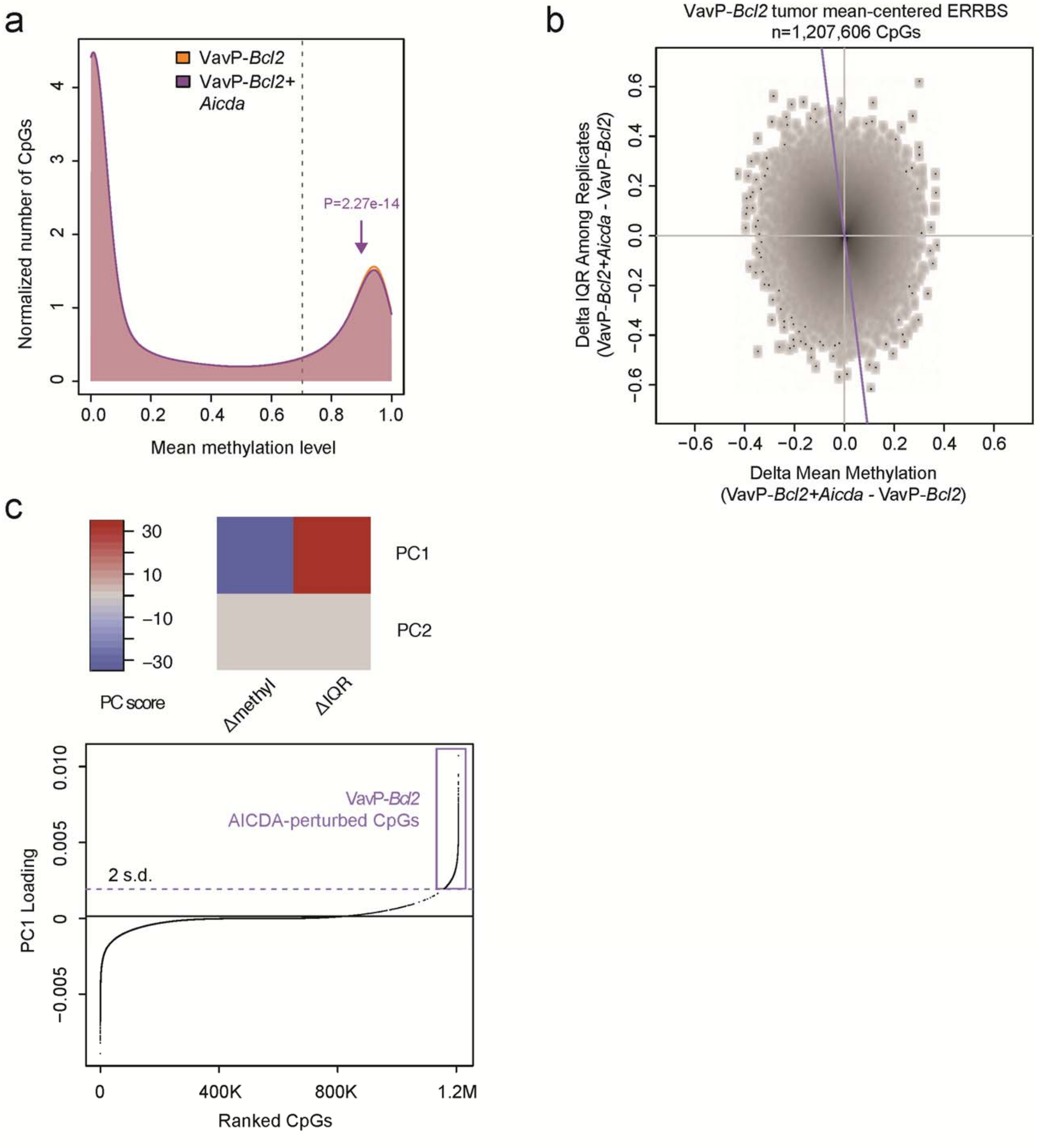

Supplementary Figure 2. AICDA alters methylome via hypomethylation and increased inter-tumor heterogeneiety in VavP-*Bcl2*+*Aicda* lymphomas. (a) Density plot showing the mean methylation distribution of all CpGs respresented in the VavP-*Bcl2* (n=6) and VavP-*Bcl2*+*Aicda* (n=7) ERRBS profiles. Overexpression of AICDA is associated with fewer highly methylated CpGs, Fisher's Exact Test (b) Density scatterplot showing centered data representing change in mean methylation level versus change in IQR between VavP-*Bcl2* and VavP-*Bcl2*+*Aicda* for all ERRBS-represented CpGs. The purple line indicates the principal component axis. (c) Principal component decomposition of centerend data from (b). Top, PC scores indicating methylation pattern comprising decreased methylation level and increased inter-tumor diversity withn VavP-*Bcl2*+*Aicda* tumors. Bottom, rnaked CpG componetn loading factors. Horizontal black line indicates mean loading factor and purple rectangle indicates AICDA perturbation CpG signature.

a

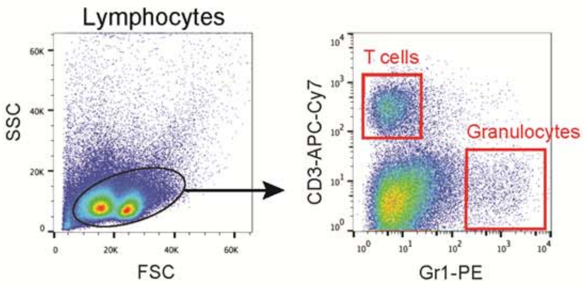

Sample Number	Sample Name	Sample Type	Number of sorted cells	Total DNA amount(ng)
1	VavP-*Bcl2*_1, Gr1 cells	Granulocytes	63000	88
2	VavP-*Bcl2*_2, Gr1 cells	Granulocytes	197000	216
3	VavP-*Bcl2*_3 Gr1 cells	Granulocytes	50000	38.8
4	VavP-*Bcl2+Aicda*_1, Gr1 cells	Granulocytes	110000	107.6
5	VavP-*Bcl2+Aicda*_2, Gr1 cells	Granulocytes	90000	147.6
6	VavP-*Bcl2+Aicda*_3, Gr1 cells	Granulocytes	245000	672
7	VavP-*Bcl2*_1 T cells	T cells	277000	346
8	VavP-*Bcl2*_2, T cells	T cells	754000	1020
9	VavP-*Bcl2*_3, T cells	T cells	41000	36.6
10	VavP-*Bcl2+Aicda*_1, T cells	T cells	229000	364
11	VavP-*Bcl2+Aicda*_2, T cells	T cells	141000	142
12	VavP-*Bcl2+Aicda*_3, T cells	T cells	134000	167.2

b

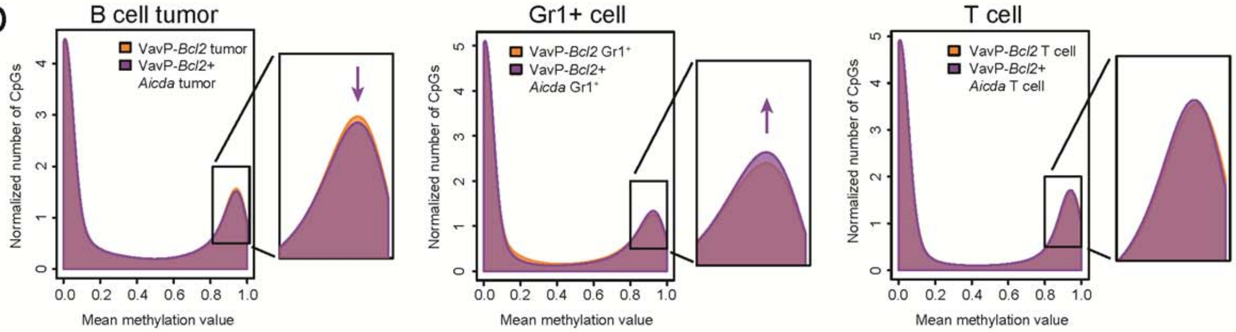

c

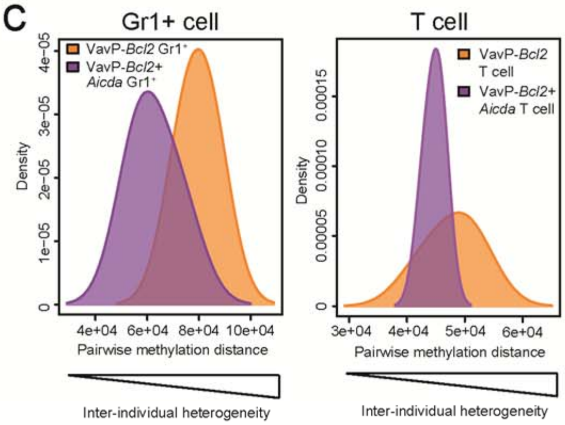

Supplementary Figure 3. Non-B cell types in VavP-Bcl2 mice do not show reduction of methylated cytosines or increase in methylation heterogeneity. (a) Flow cytometry gating strategy to isolate T cells and Gr1+ cells from the spleen of VavP-Bcl2 and VavP-Bcl2+Aicda mice. The number of sorted cells and the amount of gDNA obtained are also shown. (b) Density plots showing the mean methylation values in murine tumors (left), Gr1+ cells (middle), and T cells (right). Inset magnifies the density at highly methylated CpGs (>80%). (c) Density plot showing pairwise methylation distance for Gr1+ cells (left) and T cells (right).

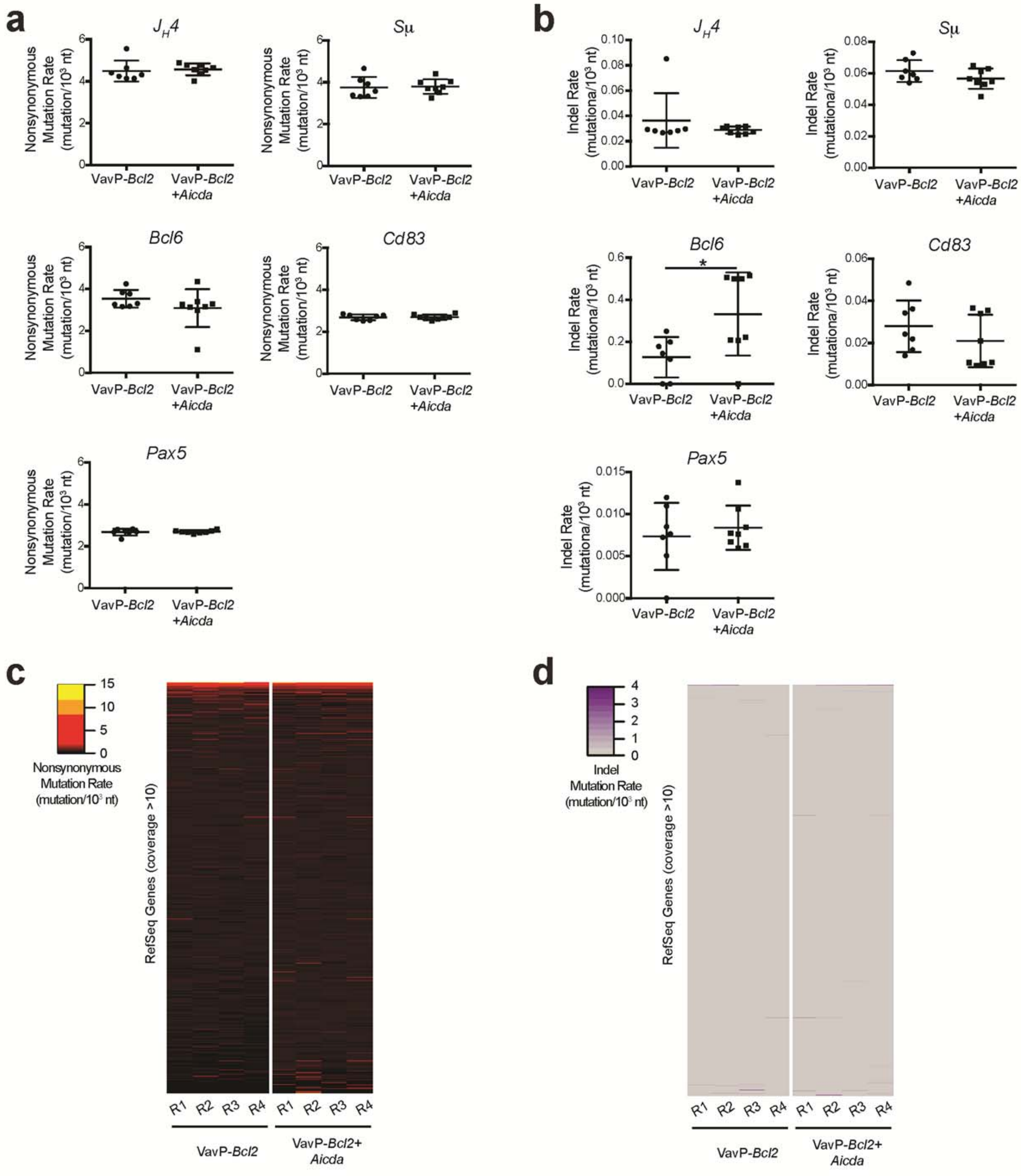

Supplementary Figure 4. AICDA off target activity in B-cells from BCL2-driven lymphomas. (a) Analysis of the nonsynonymous mutation rate (mutation/10^3 nt) in AICDA target genes (JH4, Sμ, Bcl6, Cd83 and Pax5) in B220+ cells isolated from the spleens of VavP-*Bcl2* (n=7) and VavP-*Bcl2+Aicda* (n=8) mice. Dot plots represent mean of non-synonymous mutations and error bars indicate standard deviation. (b) Analysis of the indel mutation rate (mutation/10^3 nt) in AICDA target genes (JH4, Sμ, Bcl6, Cd83 and Pax5) in B220+ cells isolated from the spleens of VavP-*Bcl2* (n=7) and VavP-*Bcl2+Aicda* (n=8) mice. Dot plots represent mean of indels and error bars indicate standard deviation; two-sided t-test, * $P < 0.05$. (c) Heat map showing the nonsynonymous mutation rates in VavP-*Bcl2* and VavP-*Bcl2+Aicda* lymphomas. No significant differences in mutation burden are observed between the two conditions; two-sided Wilcoxon signed-rank test. (d) Heat map showing the indel mutation rates in VavP-Bcl2 and VavP-Bcl2+Aicda lymphomas. No significant differences in mutation burden are observed between the two conditions; two-sided Wilcoxon signed-rank test.

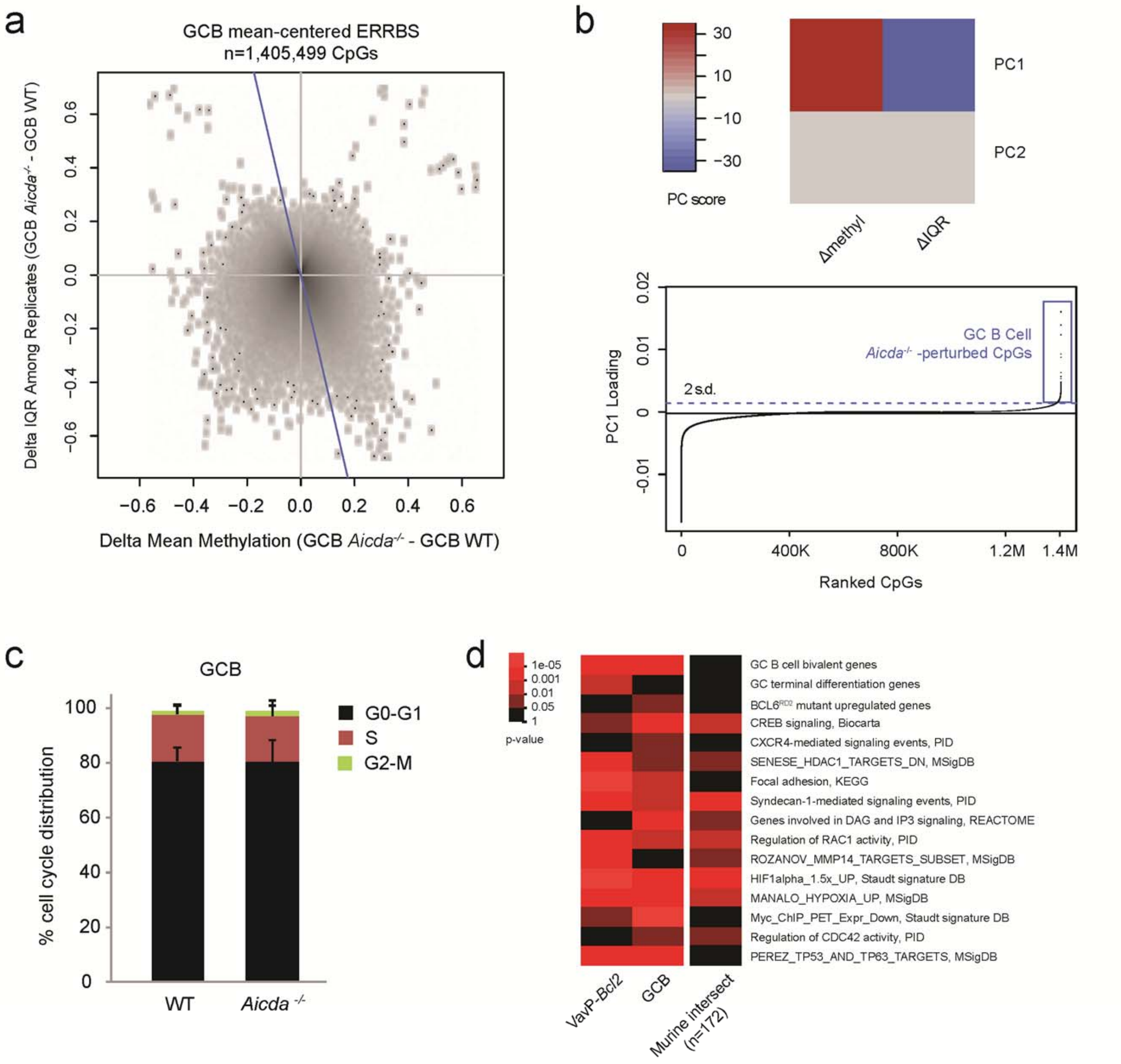

Supplementary Figure 5. Loss of AICDA in GC B-cells results in hypermethylation and decreased inter-tumor heterogeneity. (a) Density scatterplot showing centered data representing change in mean methylation level versus change in IQR between *Aicda*$^{-/-}$ and wild-type GC B-cells. The blue line indicates the principal component axis. (b) Principal component decomposition of centered data from (a). Top, PC scores indicating methylation pattern comprised of increase in methylation and decrease in inter-individual heterogeneity within *Aicda*$^{-/-}$ GC B-cells relative to wild-type controls. Bottom, ranked CpG component loading factors. Horizontal black line indicates mean loading factor and blue rectangle indicates AICDA perturbation CpG signature. (c) Percentages of cells in G0/G1, S and G2/M phases, detected by BrdU incorporation and 7-AAD staining in WT (n=5) and *Aicda*$^{-/-}$ (n=5) GC B cells. (d) Heatmap showing enrichment significance of AICDA-perturbed genes for signatures identified by various databases; hypergeometric test.

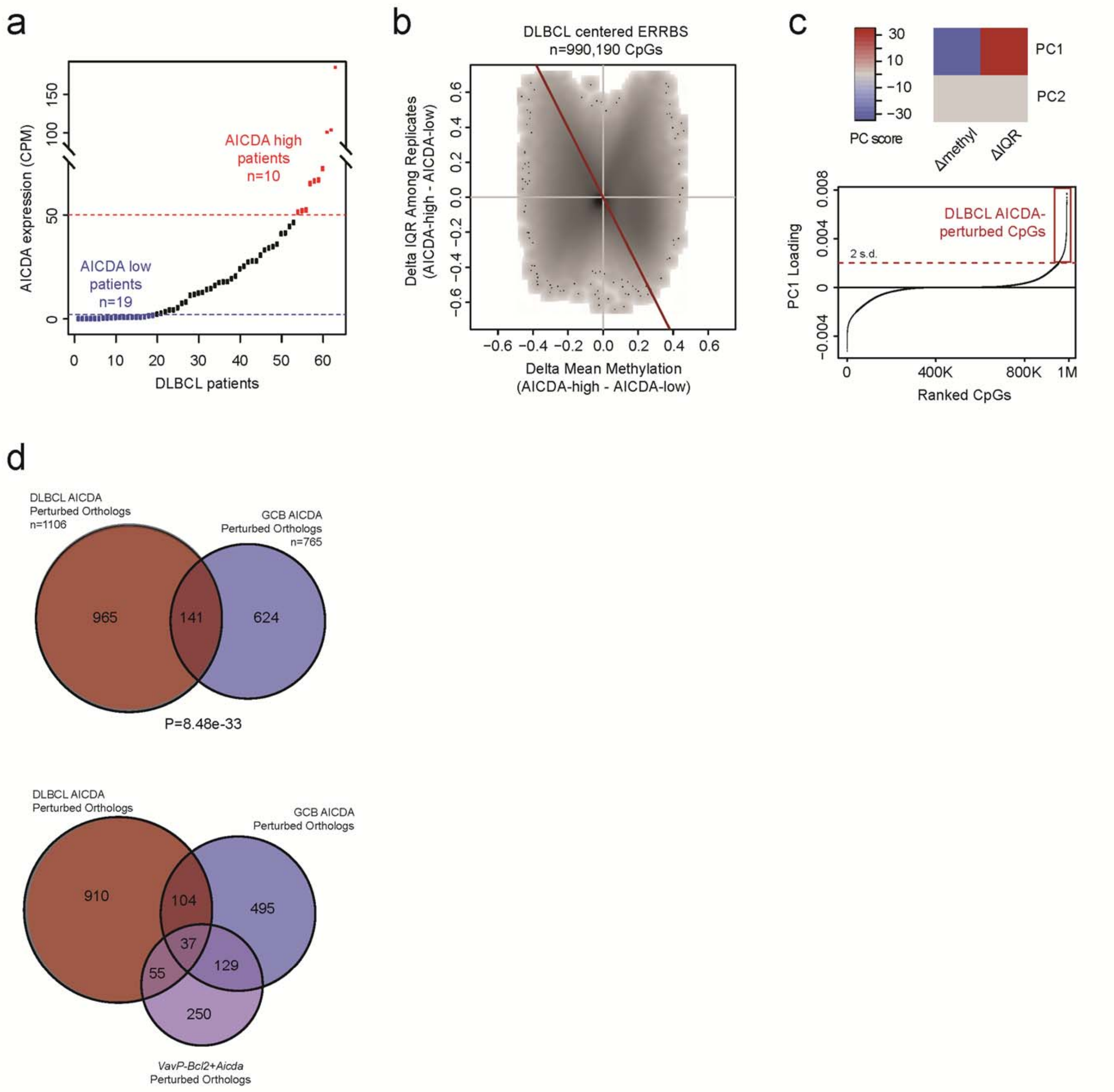

Supplementary Figure 6. High AICDA expression in DLBCLs is associated with increased DNA methylation inter-tumor heterogeneity and hypomethylation. (a) Expression of AICDA within DLBCL cohort. Cases with AICDA expression <2 CPM were characterized as low expressors (AICDA-low) and cases with expression >50 CPM were characterized as high expressors (AICDA-high). (b) Density scatterplot showing centered data representing change in mean methylation level versus change in IQR between AICDA-low DLBCL and AICDA-high DLBCL. The red line indicates the principal component axis. (c) Principal component decomposition of centered data from (b). Top, PC scores indicating methylation pattern comprising decrease in methylation and increase in inter-tumor heterogeneity within AICDA-high DLBCL. Bottom, ranked CpG component loading factors. Horizontal black line indicates mean loading factor and red rectangle indicates AICDA perturbation CpG signature. (d) Venn diagrams showing the overlap between gene orthologs significantly over-representing DLBCL AICDA-perturbed CpGs and murine orthologs over-representing GC *Aicda*$^{-/-}$ perturbed CpGs (top) or between orthologs over-representing VavP-*Bcl2*+*Aicda* signature CpGs and GC *Aicda*$^{-/-}$ signature CpGs (bottom); hypergeometric test.

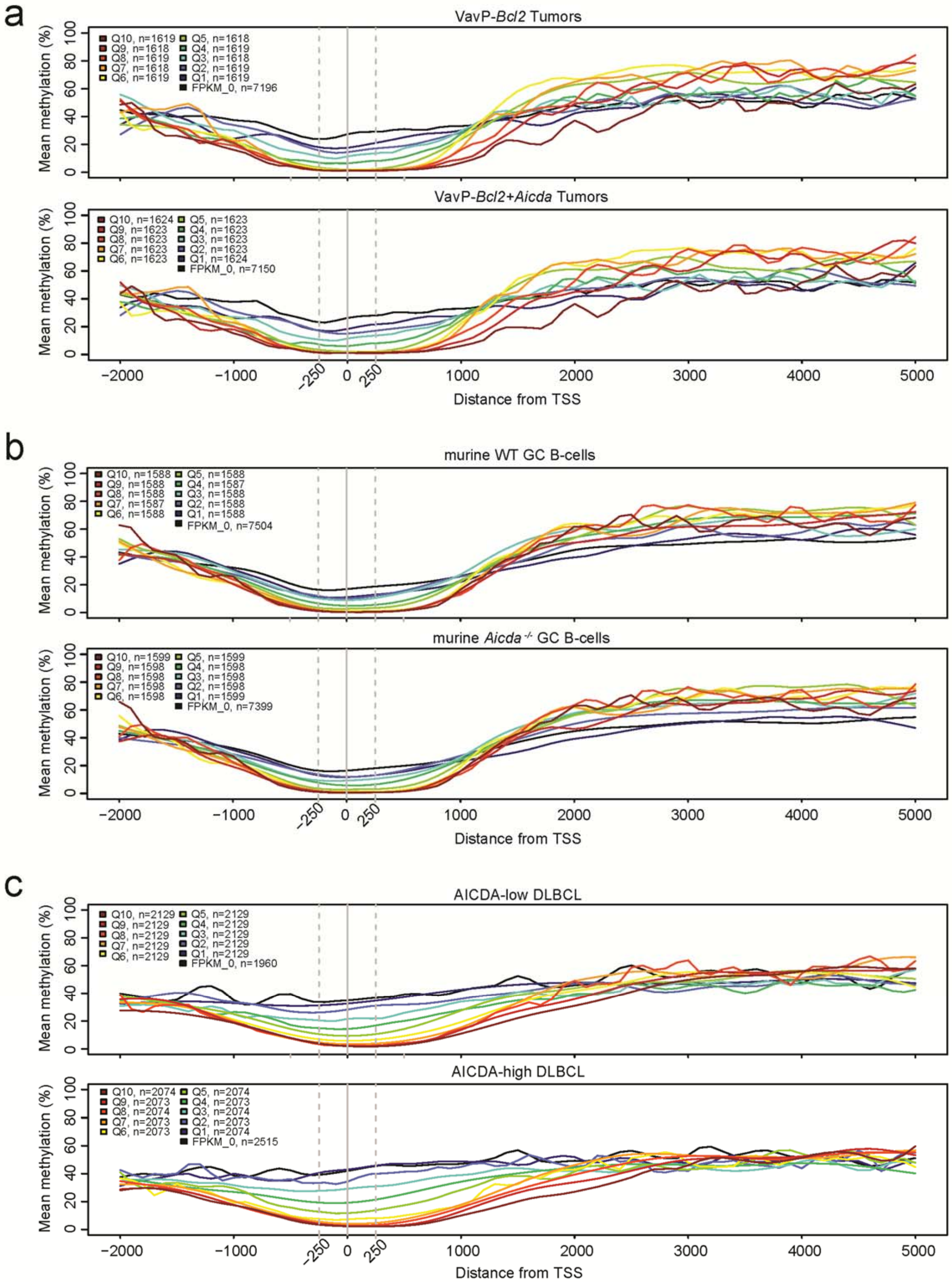

Supplementary Figure 7. Cytosine methylation at TSS is associated with low gene expression, but not with AICDA-associated gene expression changes. (a) Plots showing mean cytosine methylation of gene promoters according to their expression in VavP-*Bcl2* tumors (top) and VavP-*Bcl2*+*Aicda* tumors (bottom); "FPKM_0" indicates no expression, Q1-Q10 indicates deciles of expression from lowest to highest. (b) Plot showing mean cytosine methylation of gene promoters according to their expression in WTGC B cells (top) and *Aicda* $^{-/-}$ GC B cells (bottom). (c) Plot showing mean cytosine methylation of gene promoters according to their expression in AICDA-low (top) and AICDA-high DLBCL (bottom).

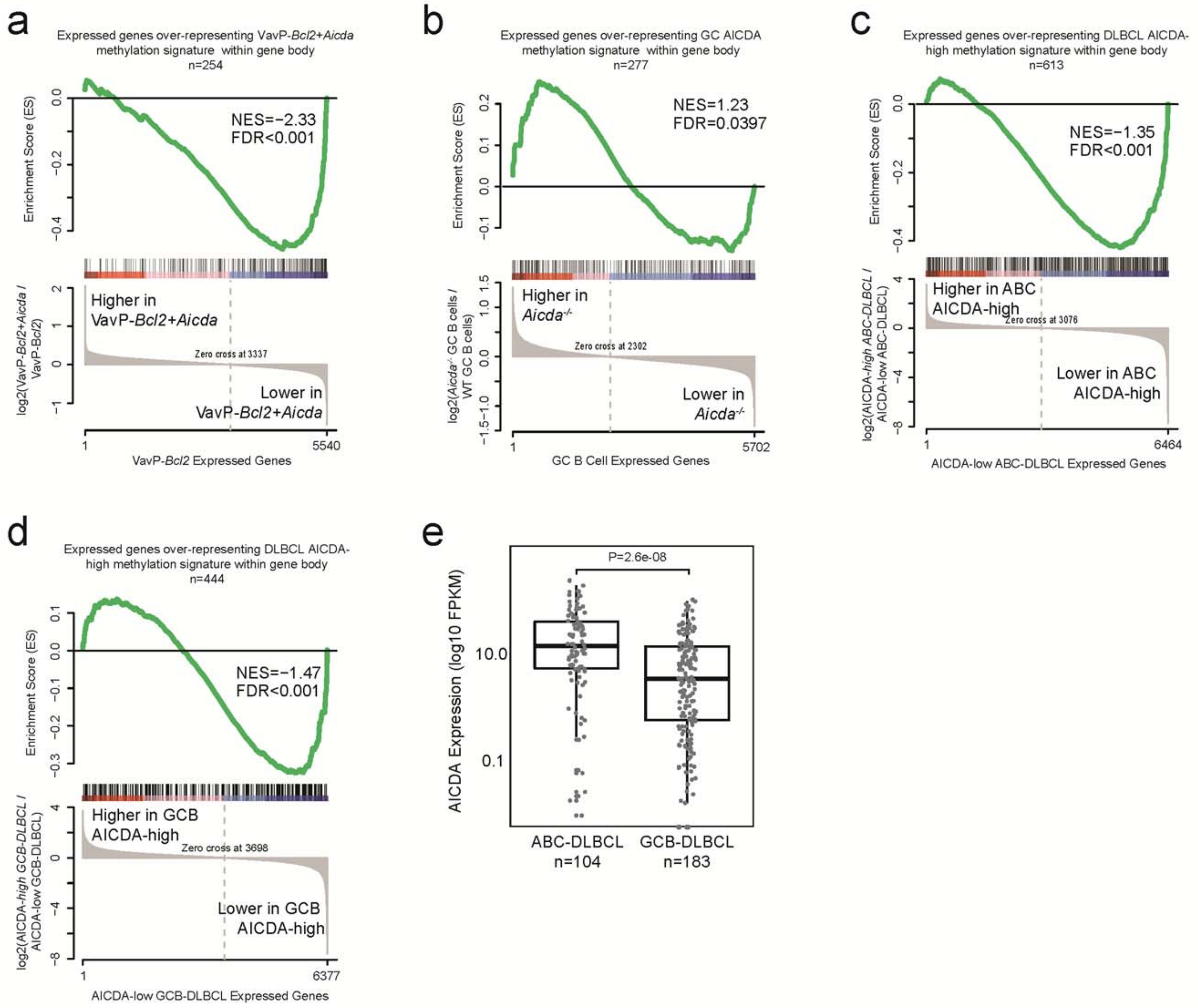

Supplementary Figure 8. AICDA perturbed genes are associated with decreased gene expression. (a) GSEA in VavP-*Bcl2+Aicda* and VavP-*Bcl2* tumors using genes significantly overrepresenting VavP-*Bcl2+Aicda* signature CpGs within their gene bodies. To remove noise, only genes expressed in VavP-*Bcl2* controls (FPKM>5) were assessed. (b) GSEA in *Aicda*$^{-/-}$ and WT GC B-cells using genes significantly overrepresenting GC AICDA signature CpGs within gene bodies. Only genes expressed in WT GC B-cell controls (FPKM>5) were assessed. (c) GSEA in AICDA-high and AICDA-low ABC-DLBCL cases using genes significantly overrepresenting DLBCL AICDA signature CpGs within gene bodies. Only genes expressed in AICDA-low ABC-DLBCL controls were assessed. (d) GSEA in AICDA-high and AICDA-low GCB-DLBCL cases using genes significantly overrepresenting DLBCL AICDA signature CpGs within gene bodies. Only genes expressed in AICDA-low GCB-DLBCL controls were assessed. (e) Box plot showing the expression of AICDA among ABC- and GCB-DLBCL cases; WIlcoxon rank-sum test.

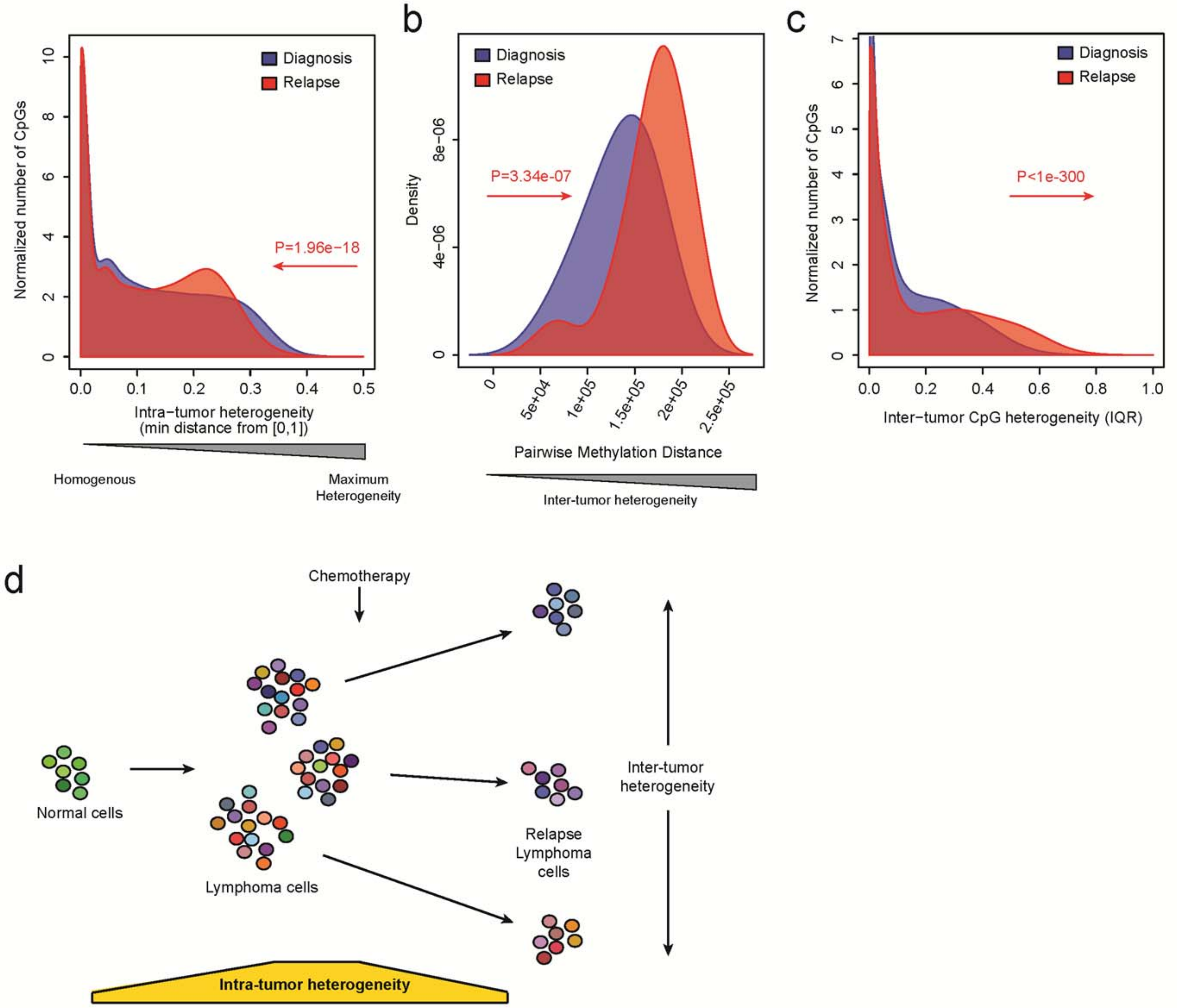

Supplementary Figure 9. Relapsed DLBCL have decreased intra-tumor heterogeneity and increased inter-tumor heterogeneity compared to diagnosis. (a) Density plot showing mean intra-tumor heterogeneity of glo bal diagnosis and relapse DLBCL ERRBS profiles. Intra-tumor heterogeneity density is shifted to the left, indicating a reduction compared to diagnosis. (b-c) Density plot showing pairwise methylation distance of DLBCL profiles (b) and IQR of all commonly represented CpGs among respective diagnosis and relapsed profiles (c). In both cases, the density is shifted to the right, indicating an increase in inter-tumor heterogeneity. (d) Illustration depicting how lymphoma cells are more heterogeneous than normal cells and exhibit both intra- and inter-tumor heterogeneity, whereas at
