# Supplementary material for: AICDA drives epigenetic heterogeneity and accelerates germinal center-derived lymphomagenesis
Source: Nat Commun. 2018 Jan 15;9:222. doi: 10.1038/s41467-017-02595-w (PMC5768781; doi:10.1038/s41467-017-02595-w)
Supplement: Supplementary file 3 — Description of Additional Supplementary Files [file 41467_2017_2595_MOESM3_ESM.pdf]

Description of Additional Supplementary Files

File Name: Supplementary Data 1
Description: Genes over-representing AICDA-perturbed CpG signature.

File Name: Supplementary Data 2
Description: Leading edge genes showing AICDA-perturbed, down-regulated genes in mouse and human lymphoma.
