# Supplementary material for: AICDA drives epigenetic heterogeneity and accelerates germinal center-derived lymphomagenesis
Source: Nat Commun. 2018 Jan 15;9:222. doi: 10.1038/s41467-017-02595-w (PMC5768781; doi:10.1038/s41467-017-02595-w)
Supplement: Supplementary file 4 — Supplementary Data 1 [file 41467_2017_2595_MOESM4_ESM.docx]

**Supplementary Data 1. Genes over-representing AICDA-perturbed CpG signature**

| Human ortholog | Murine ortholog | VavP-Bcl2 AICDA-perturbed | GC B cell AICDA-perturbed | Human DLBCL AICDA-perturbed |
| --- | --- | --- | --- | --- |
| ZNF423 | Zfp423 | Y | Y | Y |
| WWC1 | Wwc1 | Y | Y | Y |
| SMAD6 | Smad6 | Y | Y | Y |
| SLC7A7 | Slc7a7 | Y | Y | Y |
| SHANK3 | Shank3 | Y | Y | Y |
| SCUBE1 | Scube1 | Y | Y | Y |
| SCARA5 | Scara5 | Y | Y | Y |
| RIN2 | Rin2 | Y | Y | Y |
| RGS3 | Rgs3 | Y | Y | Y |
| PTK7 | Ptk7 | Y | Y | Y |
| PLCH2 | Plch2 | Y | Y | Y |
| OBSCN | Obscn | Y | Y | Y |
| NMNAT2 | Nmnat2 | Y | Y | Y |
| MCF2L | Mcf2l | Y | Y | Y |
| LTBP3 | Ltbp3 | Y | Y | Y |
| LAMA5 | Lama5 | Y | Y | Y |
| KIF1A | Kif1a | Y | Y | Y |
| JPH3 | Jph3 | Y | Y | Y |
| GRIK4 | Grik4 | Y | Y | Y |
| GPR162 | Gpr162 | Y | Y | Y |
| GABBR2 | Gabbr2 | Y | Y | Y |
| EZR | Ezr | Y | Y | Y |
| EVPL | Evpl | Y | Y | Y |
| EPHB2 | Ephb2 | Y | Y | Y |
| DYSF | Dysf | Y | Y | Y |
| DSCAML1 | Dscaml1 | Y | Y | Y |
| DOCK6 | Dock6 | Y | Y | Y |
| DNAH17 | Dnahc17 | Y | Y | Y |
| CUX2 | Cux2 | Y | Y | Y |
| COL5A1 | Col5a1 | Y | Y | Y |
| COL23A1 | Col23a1 | Y | Y | Y |
| CHD5 | Chd5 | Y | Y | Y |
| CDH23 | Cdh23 | Y | Y | Y |
| CAMTA1 | Camta1 | Y | Y | Y |
| CACNB1 | Cacnb1 | Y | Y | Y |
| CACNA1G | Cacna1g | Y | Y | Y |
| ADCY5 | Adcy5 | Y | Y | Y |
| ZNF839 | Zfp839 | Y | Y | - |
| ZNF536 | Zfp536 | Y | Y | - |
| ZNF362 | Zfp362 | Y | Y | - |
| ZBTB7C | Zbtb7c | Y | Y | - |
| ZBTB22 | Zbtb22 | Y | Y | - |
| ZBTB16 | Zbtb16 | Y | Y | - |
| WWOX | Wwox | Y | Y | - |
| VPS8 | Vps8 | Y | Y | - |
| TUBB | Tubb5 | Y | Y | - |
| TSPAN18 | Tspan18 | Y | Y | - |
| TRPC7 | Trpc7 | Y | Y | - |
| TMPRSS9 | Tmprss9 | Y | Y | - |
| TMEM132D | Tmem132d | Y | Y | - |
| TMEM132C | Tmem132c | Y | Y | - |
| TMEM121 | Tmem121 | Y | Y | - |
| TMEM117 | Tmem117 | Y | Y | - |
| TMEM108 | Tmem108 | Y | Y | - |
| TIMP2 | Timp2 | Y | Y | - |
| TEAD1 | Tead1 | Y | Y | - |
| TBC1D30 | Tbc1d30 | Y | Y | - |
| SVOP | Svop | Y | Y | - |
| SULF1 | Sulf1 | Y | Y | - |
| SPRED2 | Spred2 | Y | Y | - |
| SLIT3 | Slit3 | Y | Y | - |
| SLC28A3 | Slc28a3 | Y | Y | - |
| SEZ6L | Sez6l | Y | Y | - |
| SEMA5B | Sema5b | Y | Y | - |
| SEL1L3 | Sel1l3 | Y | Y | - |
| SDK2 | Sdk2 | Y | Y | - |
| SCN5A | Scn5a | Y | Y | - |
| RUSC2 | Rusc2 | Y | Y | - |
| RTKN2 | Rtkn2 | Y | Y | - |
| RPS6KL1 | Rps6kl1 | Y | Y | - |
| RNFT2 | Rnft2 | Y | Y | - |
| RNF165 | Rnf165 | Y | Y | - |
| RBPMS | Rbpms | Y | Y | - |
| RBFOX3 | Rbfox3 | Y | Y | - |
| RBFOX1 | Rbfox1 | Y | Y | - |
| RASGRF1 | Rasgrf1 | Y | Y | - |
| PTPRT | Ptprt | Y | Y | - |
| PTPRN2 | Ptprn2 | Y | Y | - |
| PTPRG | Ptprg | Y | Y | - |
| PTPN14 | Ptpn14 | Y | Y | - |
| PRKAR1B | Prkar1b | Y | Y | - |
| PRDM16 | Prdm16 | Y | Y | - |
| PLEKHH2 | Plekhh2 | Y | Y | - |
| PDZD7 | Pdzd7 | Y | Y | - |
| PCNXL2 | Pcnxl2 | Y | Y | - |
| PCDHGB5 | Pcdhgb5 | Y | Y | - |
| PCDHGB4 | Pcdhgb4 | Y | Y | - |
| PCDHGB2 | Pcdhgb2 | Y | Y | - |
| PCDHGB1 | Pcdhgb1 | Y | Y | - |
| PCDHGA8 | Pcdhga8 | Y | Y | - |
| PCDHGA7 | Pcdhga7 | Y | Y | - |
| PCDHGA6 | Pcdhga6 | Y | Y | - |
| PCDHGA5 | Pcdhga5 | Y | Y | - |
| PCDHGA4 | Pcdhga4 | Y | Y | - |
| PCDHGA3 | Pcdhga3 | Y | Y | - |
| PCDHGA2 | Pcdhga2 | Y | Y | - |
| PCDHGA1 | Pcdhga1 | Y | Y | - |
| NA | Pcdha4-g | Y | Y | - |
| PANX2 | Panx2 | Y | Y | - |
| NTM | Ntm | Y | Y | - |
| NPTX2 | Nptx2 | Y | Y | - |
| NPAS2 | Npas2 | Y | Y | - |
| NFIX | Nfix | Y | Y | - |
| NECAB2 | Necab2 | Y | Y | - |
| MTUS2 | Mtus2 | Y | Y | - |
| MN1 | Mn1 | Y | Y | - |
| MINPP1 | Minpp1 | Y | Y | - |
| MFHAS1 | Mfhas1 | Y | Y | - |
| LOXHD1 | Loxhd1 | Y | Y | - |
| LHFPL3 | Lhfpl3 | Y | Y | - |
| L3MBTL1 | L3mbtl1 | Y | Y | - |
| KSR2 | Ksr2 | Y | Y | - |
| KIF6 | Kif6 | Y | Y | - |
| KDM4A | Kdm4a | Y | Y | - |
| KALRN | Kalrn | Y | Y | - |
| ITPKA | Itpka | Y | Y | - |
| IL17D | Il17d | Y | Y | - |
| IGSF21 | Igsf21 | Y | Y | - |
| NA | Ifnlr1 | Y | Y | - |
| HYDIN | Hydin | Y | Y | - |
| HOXB3 | Hoxb3 | Y | Y | - |
| GRIP2 | Grip2 | Y | Y | - |
| GRIK3 | Grik3 | Y | Y | - |
| GNAS | Gnas | Y | Y | - |
| NA | Gm6878 | Y | Y | - |
| GLP1R | Glp1r | Y | Y | - |
| GLIS1 | Glis1 | Y | Y | - |
| GALNTL1 | Galntl1 | Y | Y | - |
| FRMPD1 | Frmpd1 | Y | Y | - |
| FNDC3B | Fndc3b | Y | Y | - |
| FNBP1 | Fnbp1 | Y | Y | - |
| FAM171A2 | Fam171a2 | Y | Y | - |
| ESRRB | Esrrb | Y | Y | - |
| EPHA8 | Epha8 | Y | Y | - |
| EML1 | Eml1 | Y | Y | - |
| EBF2 | Ebf2 | Y | Y | - |
| EBF1 | Ebf1 | Y | Y | - |
| DST | Dst | Y | Y | - |
| DNAJC16 | Dnajc16 | Y | Y | - |
| DNAH10 | Dnahc10 | Y | Y | - |
| DLGAP2 | Dlgap2 | Y | Y | - |
| DACT3 | Dact3 | Y | Y | - |
| CRYBB1 | Crybb1 | Y | Y | - |
| CRB2 | Crb2 | Y | Y | - |
| COL4A1 | Col4a1 | Y | Y | - |
| COL13A1 | Col13a1 | Y | Y | - |
| C1orf94 | CK137956 | Y | Y | - |
| CILP2 | Cilp2 | Y | Y | - |
| CDK14 | Cdk14 | Y | Y | - |
| CDH4 | Cdh4 | Y | Y | - |
| CDH22 | Cdh22 | Y | Y | - |
| CAPN11 | Capn11 | Y | Y | - |
| CAMK2A | Camk2a | Y | Y | - |
| CADM1 | Cadm1 | Y | Y | - |
| CACNA2D3 | Cacna2d3 | Y | Y | - |
| CACNA1I | Cacna1i | Y | Y | - |
| CACNA1C | Cacna1c | Y | Y | - |
| BSN | Bsn | Y | Y | - |
| BRPF1 | Brpf1 | Y | Y | - |
| BCL11B | Bcl11b | Y | Y | - |
| ASPH | Asph | Y | Y | - |
| ARVCF | Arvcf | Y | Y | - |
| ARSG | Arsg | Y | Y | - |
| APBB2 | Apbb2 | Y | Y | - |
| ANKS1B | Anks1b | Y | Y | - |
| AKR7A2 | Akr7a5 | Y | Y | - |
| AJAP1 | Ajap1 | Y | Y | - |
| ADAMTS17 | Adamts17 | Y | Y | - |
| NA | A930011G23Rik | Y | Y | - |
| C10orf71 | 3425401B19Rik | Y | Y | - |
| NA | 2810410L24Rik | Y | Y | - |
| NA | 2310005E17Rik | Y | Y | - |
| XIRP1 | Xirp1 | Y | - | Y |
| WNT9A | Wnt9a | Y | - | Y |
| USP2 | Usp2 | Y | - | Y |
| TTLL10 | Ttll10 | Y | - | Y |
| TNFAIP2 | Tnfaip2 | Y | - | Y |
| THSD4 | Thsd4 | Y | - | Y |
| TET1 | Tet1 | Y | - | Y |
| TEAD4 | Tead4 | Y | - | Y |
| SLC6A11 | Slc6a11 | Y | - | Y |
| SLC4A1 | Slc4a1 | Y | - | Y |
| SEMA6B | Sema6b | Y | - | Y |
| SEMA4D | Sema4d | Y | - | Y |
| RREB1 | Rreb1 | Y | - | Y |
| ROBO1 | Robo1 | Y | - | Y |
| RNF144A | Rnf144a | Y | - | Y |
| RHBDF2 | Rhbdf2 | Y | - | Y |
| RAB36 | Rab36 | Y | - | Y |
| PXN | Pxn | Y | - | Y |
| PTPN3 | Ptpn3 | Y | - | Y |
| PTOV1 | Ptov1 | Y | - | Y |
| PRICKLE2 | Prickle2 | Y | - | Y |
| PPP6R3 | Ppp6r3 | Y | - | Y |
| PLXNB1 | Plxnb1 | Y | - | Y |
| PIK3R5 | Pik3r5 | Y | - | Y |
| PDE9A | Pde9a | Y | - | Y |
| PARD3 | Pard3 | Y | - | Y |
| P2RX5 | P2rx5 | Y | - | Y |
| MGAT5B | Mgat5b | Y | - | Y |
| LAMC3 | Lamc3 | Y | - | Y |
| KLF7 | Klf7 | Y | - | Y |
| KIRREL3 | Kirrel3 | Y | - | Y |
| KCNH1 | Kcnh1 | Y | - | Y |
| KAZN | Kazn | Y | - | Y |
| IL6R | Il6ra | Y | - | Y |
| HLCS | Hlcs | Y | - | Y |
| GRK5 | Grk5 | Y | - | Y |
| GDPD5 | Gdpd5 | Y | - | Y |
| FHOD3 | Fhod3 | Y | - | Y |
| FGFR1 | Fgfr1 | Y | - | Y |
| ERBB2 | Erbb2 | Y | - | Y |
| DNAH2 | Dnahc2 | Y | - | Y |
| DNAH1 | Dnahc1 | Y | - | Y |
| DIP2C | Dip2c | Y | - | Y |
| DAGLA | Dagla | Y | - | Y |
| CTIF | Ctif | Y | - | Y |
| COL18A1 | Col18a1 | Y | - | Y |
| COL16A1 | Col16a1 | Y | - | Y |
| CDC42BPB | Cdc42bpb | Y | - | Y |
| CCDC27 | Ccdc27 | Y | - | Y |
| CACNA2D4 | Cacna2d4 | Y | - | Y |
| CABLES1 | Cables1 | Y | - | Y |
| BAI2 | Bai2 | Y | - | Y |
| ARL3 | Arl3 | Y | - | Y |
| ALDH1A3 | Aldh1a3 | Y | - | Y |
| ACOT7 | Acot7 | Y | - | Y |
| ZMYND15 | Zmynd15 | - | Y | Y |
| ZFPM1 | Zfpm1 | - | Y | Y |
| ZFHX2 | Zfhx2 | - | Y | Y |
| ZBTB48 | Zbtb48 | - | Y | Y |
| XKR6 | Xkr6 | - | Y | Y |
| VAV2 | Vav2 | - | Y | Y |
| UNC5B | Unc5b | - | Y | Y |
| TTC7A | Ttc7 | - | Y | Y |
| TP73 | Trp73 | - | Y | Y |
| TRIM8 | Trim8 | - | Y | Y |
| TRIM41 | Trim41 | - | Y | Y |
| TRANK1 | Trank1 | - | Y | Y |
| TLR9 | Tlr9 | - | Y | Y |
| TIAM2 | Tiam2 | - | Y | Y |
| THOP1 | Thop1 | - | Y | Y |
| TH | Th | - | Y | Y |
| SYN2 | Syn2 | - | Y | Y |
| SYK | Syk | - | Y | Y |
| STAB1 | Stab1 | - | Y | Y |
| SSPO | Sspo | - | Y | Y |
| SPSB1 | Spsb1 | - | Y | Y |
| SPIRE2 | Spire2 | - | Y | Y |
| SPATC1 | Spatc1 | - | Y | Y |
| SORCS2 | Sorcs2 | - | Y | Y |
| SLC6A9 | Slc6a9 | - | Y | Y |
| SLC29A4 | Slc29a4 | - | Y | Y |
| SLC23A2 | Slc23a2 | - | Y | Y |
| SKI | Ski | - | Y | Y |
| SH3RF3 | Sh3rf3 | - | Y | Y |
| SEMA3G | Sema3g | - | Y | Y |
| SCAMP4 | Scamp4 | - | Y | Y |
| SAMD11 | Samd11 | - | Y | Y |
| RXRA | Rxra | - | Y | Y |
| RAPGEF4 | Rapgef4 | - | Y | Y |
| RADIL | Radil | - | Y | Y |
| PRRC2B | Prrc2b | - | Y | Y |
| PRKCZ | Prkcz | - | Y | Y |
| PRKCA | Prkca | - | Y | Y |
| POU2F2 | Pou2f2 | - | Y | Y |
| PEBP4 | Pebp4 | - | Y | Y |
| PCSK4 | Pcsk4 | - | Y | Y |
| PACSIN2 | Pacsin2 | - | Y | Y |
| NOTCH3 | Notch3 | - | Y | Y |
| NFIC | Nfic | - | Y | Y |
| NFASC | Nfasc | - | Y | Y |
| NAV2 | Nav2 | - | Y | Y |
| MVD | Mvd | - | Y | Y |
| MMP11 | Mmp11 | - | Y | Y |
| MGMT | Mgmt | - | Y | Y |
| MFNG | Mfng | - | Y | Y |
| MBP | Mbp | - | Y | Y |
| MAPT | Mapt | - | Y | Y |
| MACROD1 | Macrod1 | - | Y | Y |
| LRRN2 | Lrrn2 | - | Y | Y |
| LRP5 | Lrp5 | - | Y | Y |
| LRP1 | Lrp1 | - | Y | Y |
| KLHL29 | Klhl29 | - | Y | Y |
| KIFC3 | Kifc3 | - | Y | Y |
| KIF26B | Kif26b | - | Y | Y |
| KCNT1 | Kcnt1 | - | Y | Y |
| KCNQ1 | Kcnq1 | - | Y | Y |
| ITGB4 | Itgb4 | - | Y | Y |
| ITGA11 | Itga11 | - | Y | Y |
| HSPG2 | Hspg2 | - | Y | Y |
| GRM4 | Grm4 | - | Y | Y |
| GRHPR | Grhpr | - | Y | Y |
| GPC1 | Gpc1 | - | Y | Y |
| GNG7 | Gng7 | - | Y | Y |
| FAM69B | Fam69b | - | Y | Y |
| FAM65C | Fam65c | - | Y | Y |
| FAM178B | Fam178b | - | Y | Y |
| ESPNL | Espnl | - | Y | Y |
| EFNA2 | Efna2 | - | Y | Y |
| DTX1 | Dtx1 | - | Y | Y |
| DPF3 | Dpf3 | - | Y | Y |
| CTBP2 | Ctbp2 | - | Y | Y |
| CSRNP1 | Csrnp1 | - | Y | Y |
| CRHR1 | Crhr1 | - | Y | Y |
| CORO2A | Coro2a | - | Y | Y |
| COL7A1 | Col7a1 | - | Y | Y |
| CLNK | Clnk | - | Y | Y |
| CDHR5 | Cdhr5 | - | Y | Y |
| CDHR2 | Cdhr2 | - | Y | Y |
| CDC42BPG | Cdc42bpg | - | Y | Y |
| CCDC85C | Ccdc85c | - | Y | Y |
| CCDC33 | Ccdc33 | - | Y | Y |
| CASZ1 | Casz1 | - | Y | Y |
| CARD10 | Card10 | - | Y | Y |
| CAMK2B | Camk2b | - | Y | Y |
| CACNA2D2 | Cacna2d2 | - | Y | Y |
| C2 | C2 | - | Y | Y |
| BEGAIN | Begain | - | Y | Y |
| ATP2B2 | Atp2b2 | - | Y | Y |
| ASS1 | Ass1 | - | Y | Y |
| ARRB1 | Arrb1 | - | Y | Y |
| ARHGEF19 | Arhgef19 | - | Y | Y |
| ANK3 | Ank3 | - | Y | Y |
| ADAT3 | Adat3 | - | Y | Y |
| ADAMTS14 | Adamts14 | - | Y | Y |
| ABTB2 | Abtb2 | - | Y | Y |
| ABCB9 | Abcb9 | - | Y | Y |
| KIAA1598 | 4930506M07Rik | - | Y | Y |
| 9-Sep | 9-Sep | - | Y | Y |
| 5-Sep | 5-Sep | - | Y | Y |
| ZPLD1 | Zpld1 | Y | - | - |
| ZFYVE27 | Zfyve27 | Y | - | - |
| ZNF366 | Zfp366 | Y | - | - |
| ZFP30 | Zfp30 | Y | - | - |
| ZNF217 | Zfp217 | Y | - | - |
| XYLT1 | Xylt1 | Y | - | - |
| XKR4 | Xkr4 | Y | - | - |
| XIRP2 | Xirp2 | Y | - | - |
| WWC2 | Wwc2 | Y | - | - |
| WTIP | Wtip | Y | - | - |
| WNT3A | Wnt3a | Y | - | - |
| WNK4 | Wnk4 | Y | - | - |
| VWF | Vwf | Y | - | - |
| VWA5B1 | Vwa5b1 | Y | - | - |
| NA | Vmn2r81 | Y | - | - |
| NA | Vmn2r59 | Y | - | - |
| VIPR1 | Vipr1 | Y | - | - |
| VEGFC | Vegfc | Y | - | - |
| UNC119B | Unc119b | Y | - | - |
| UBE2QL1 | Ube2ql1 | Y | - | - |
| TTLL3 | Ttll3 | Y | - | - |
| TTLL11 | Ttll11 | Y | - | - |
| TSPAN15 | Tspan15 | Y | - | - |
| TSHZ2 | Tshz2 | Y | - | - |
| TRPM3 | Trpm3 | Y | - | - |
| TRPM1 | Trpm1 | Y | - | - |
| TF | Trf | Y | - | - |
| TOX2 | Tox2 | Y | - | - |
| TNXB | Tnxb | Y | - | - |
| TNIK | Tnik | Y | - | - |
| TMPRSS4 | Tmprss4 | Y | - | - |
| TMEM44 | Tmem44 | Y | - | - |
| TMEM233 | Tmem233 | Y | - | - |
| TMEM200C | Tmem200c | Y | - | - |
| TMEM119 | Tmem119 | Y | - | - |
| TMEM100 | Tmem100 | Y | - | - |
| TMCC3 | Tmcc3 | Y | - | - |
| TLN2 | Tln2 | Y | - | - |
| TGIF2 | Tgif2 | Y | - | - |
| NA | Tenm4 | Y | - | - |
| NA | Tenm3 | Y | - | - |
| TDRD1 | Tdrd1 | Y | - | - |
| TCF4 | Tcf4 | Y | - | - |
| TBX3 | Tbx3 | Y | - | - |
| TACR1 | Tacr1 | Y | - | - |
| NA | Sult5a1 | Y | - | - |
| NA | Stpg1 | Y | - | - |
| NA | Stmnd1 | Y | - | - |
| SRRM4 | Srrm4 | Y | - | - |
| SPSB4 | Spsb4 | Y | - | - |
| SPOCK2 | Spock2 | Y | - | - |
| SPDEF | Spdef | Y | - | - |
| SPACA3 | Spaca3 | Y | - | - |
| SOX5 | Sox5 | Y | - | - |
| SORBS1 | Sorbs1 | Y | - | - |
| SNX9 | Snx9 | Y | - | - |
| SNX18 | Snx18 | Y | - | - |
| NA | Smim14 | Y | - | - |
| NA | Slc9b2 | Y | - | - |
| SLC6A4 | Slc6a4 | Y | - | - |
| SLC5A10 | Slc5a10 | Y | - | - |
| SLC39A11 | Slc39a11 | Y | - | - |
| SLC38A8 | Slc38a8 | Y | - | - |
| SLC35C1 | Slc35c1 | Y | - | - |
| SLC2A12 | Slc2a12 | Y | - | - |
| SLC26A4 | Slc26a4 | Y | - | - |
| SLC20A2 | Slc20a2 | Y | - | - |
| SLC1A7 | Slc1a7 | Y | - | - |
| SKAP1 | Skap1 | Y | - | - |
| SIRT6 | Sirt6 | Y | - | - |
| SHC2 | Shc2 | Y | - | - |
| SHANK2 | Shank2 | Y | - | - |
| SFRP1 | Sfrp1 | Y | - | - |
| SEMA7A | Sema7a | Y | - | - |
| SCRN3 | Scrn3 | Y | - | - |
| SCEL | Scel | Y | - | - |
| SALL2 | Sall2 | Y | - | - |
| RTBDN | Rtbdn | Y | - | - |
| RNF217 | Rnf217 | Y | - | - |
| RHOJ | Rhoj | Y | - | - |
| RGS9 | Rgs9 | Y | - | - |
| RGS6 | Rgs6 | Y | - | - |
| RBM20 | Rbm20 | Y | - | - |
| PTPN1 | Ptpn1 | Y | - | - |
| PTBP1 | Ptbp1 | Y | - | - |
| PPP2R2C | Ppp2r2c | Y | - | - |
| PPAN | Ppan | Y | - | - |
| POLE2 | Pole2 | Y | - | - |
| PNOC | Pnoc | Y | - | - |
| PMP22 | Pmp22 | Y | - | - |
| PLIN3 | Plin3 | Y | - | - |
| PLEKHA6 | Plekha6 | Y | - | - |
| PLB1 | Plb1 | Y | - | - |
| PKNOX2 | Pknox2 | Y | - | - |
| PIK3CB | Pik3cb | Y | - | - |
| PIAS1 | Pias1 | Y | - | - |
| PI16 | Pi16 | Y | - | - |
| PCDH17 | Pcdh17 | Y | - | - |
| PCDH10 | Pcdh10 | Y | - | - |
| PCBP3 | Pcbp3 | Y | - | - |
| PARP16 | Parp16 | Y | - | - |
| PAM | Pam | Y | - | - |
| PALMD | Palmd | Y | - | - |
| PADI4 | Padi4 | Y | - | - |
| OPN4 | Opn4 | Y | - | - |
| OFCC1 | Ofcc1 | Y | - | - |
| NTRK2 | Ntrk2 | Y | - | - |
| NTN1 | Ntn1 | Y | - | - |
| NR2F2 | Nr2f2 | Y | - | - |
| NOS3 | Nos3 | Y | - | - |
| NGLY1 | Ngly1 | Y | - | - |
| MYRIP | Myrip | Y | - | - |
| MYOM2 | Myom2 | Y | - | - |
| MYO1H | Myo1h | Y | - | - |
| MYO16 | Myo16 | Y | - | - |
| MYLK | Mylk | Y | - | - |
| MYH11 | Myh11 | Y | - | - |
| MUC4 | Muc4 | Y | - | - |
| NA | Muc19 | Y | - | - |
| NA | Mroh2a | Y | - | - |
| MMP28 | Mmp28 | Y | - | - |
| MMEL1 | Mmel1 | Y | - | - |
| NA | Mir466g | Y | - | - |
| NA | Mir3470b | Y | - | - |
| NA | Mir292 | Y | - | - |
| NA | Mir291a | Y | - | - |
| NA | Mir290 | Y | - | - |
| MAST4 | Mast4 | Y | - | - |
| MASP1 | Masp1 | Y | - | - |
| NA | Map6 | Y | - | - |
| MAP3K10 | Map3k10 | Y | - | - |
| NA | Map1a | Y | - | - |
| MAML3 | Maml3 | Y | - | - |
| MACROD2 | Macrod2 | Y | - | - |
| LRRC6 | Lrrc6 | Y | - | - |
| LRP1B | Lrp1b | Y | - | - |
| LRIG1 | Lrig1 | Y | - | - |
| LIMCH1 | Limch1 | Y | - | - |
| LGI1 | Lgi1 | Y | - | - |
| LEMD2 | Lemd2 | Y | - | - |
| LDLRAD3 | Ldlrad3 | Y | - | - |
| LAMA4 | Lama4 | Y | - | - |
| KIFC2 | Kifc2 | Y | - | - |
| KIF13A | Kif13a | Y | - | - |
| KCNQ4 | Kcnq4 | Y | - | - |
| KCNN3 | Kcnn3 | Y | - | - |
| KCNG4 | Kcng4 | Y | - | - |
| AJUBA | Jub | Y | - | - |
| JPH4 | Jph4 | Y | - | - |
| IVD | Ivd | Y | - | - |
| ITGB6 | Itgb6 | Y | - | - |
| IRF4 | Irf4 | Y | - | - |
| NA | Irf2bpl | Y | - | - |
| IQGAP2 | Iqgap2 | Y | - | - |
| INHA | Inha | Y | - | - |
| IL21 | Il21 | Y | - | - |
| IKZF2 | Ikzf2 | Y | - | - |
| IGSF3 | Igsf3 | Y | - | - |
| IGSF11 | Igsf11 | Y | - | - |
| IGF2BP1 | Igf2bp1 | Y | - | - |
| IGF1R | Igf1r | Y | - | - |
| IFNGR2 | Ifngr2 | Y | - | - |
| HPN | Hpn | Y | - | - |
| HMGA2 | Hmga2 | Y | - | - |
| HK2 | Hk2 | Y | - | - |
| GSG1L | Gsg1l | Y | - | - |
| GRIN2B | Grin2b | Y | - | - |
| GRID2 | Grid2 | Y | - | - |
| GPR161 | Gpr161 | Y | - | - |
| GPR116 | Gpr116 | Y | - | - |
| GPI | Gpi1 | Y | - | - |
| NA | Gm829 | Y | - | - |
| NA | Gm4566 | Y | - | - |
| NA | Gm16287 | Y | - | - |
| LOC100506881 | Gm13845 | Y | - | - |
| NA | Gm13446 | Y | - | - |
| NA | Gm11468 | Y | - | - |
| NA | Gm10069 | Y | - | - |
| GLTP | Gltp | Y | - | - |
| FRMD4A | Frmd4a | Y | - | - |
| FHIT | Fhit | Y | - | - |
| FGF12 | Fgf12 | Y | - | - |
| FGD6 | Fgd6 | Y | - | - |
| NA | Ffar4 | Y | - | - |
| FBXO41 | Fbxo41 | Y | - | - |
| FAM84A | Fam84a | Y | - | - |
| NA | Fam222a | Y | - | - |
| NA | Fam211b | Y | - | - |
| FAM19A5 | Fam19a5 | Y | - | - |
| NA | F730043M19Rik | Y | - | - |
| EYA4 | Eya4 | Y | - | - |
| EXT1 | Ext1 | Y | - | - |
| EXOC2 | Exoc2 | Y | - | - |
| ESR1 | Esr1 | Y | - | - |
| ERBB4 | Erbb4 | Y | - | - |
| EPS8L1 | Eps8l1 | Y | - | - |
| EPS8 | Eps8 | Y | - | - |
| EPHA6 | Epha6 | Y | - | - |
| ENOX1 | Enox1 | Y | - | - |
| ELN | Eln | Y | - | - |
| ELMOD1 | Elmod1 | Y | - | - |
| EDIL3 | Edil3 | Y | - | - |
| DSCAM | Dscam | Y | - | - |
| NA | Draxin | Y | - | - |
| DPYSL4 | Dpysl4 | Y | - | - |
| DPYS | Dpys | Y | - | - |
| DOCK5 | Dock5 | Y | - | - |
| DOCK1 | Dock1 | Y | - | - |
| DISC1 | Disc1 | Y | - | - |
| DGKZ | Dgkz | Y | - | - |
| DGKG | Dgkg | Y | - | - |
| DEXI | Dexi | Y | - | - |
| DDR1 | Ddr1 | Y | - | - |
| NA | D930007P13Rik | Y | - | - |
| CYTH3 | Cyth3 | Y | - | - |
| NA | Cyp2b13 | Y | - | - |
| CTNND2 | Ctnnd2 | Y | - | - |
| CTNNA2 | Ctnna2 | Y | - | - |
| CREB3L3 | Creb3l3 | Y | - | - |
| NA | Cpq | Y | - | - |
| CORO7 | Coro7 | Y | - | - |
| COL9A1 | Col9a1 | Y | - | - |
| COL8A1 | Col8a1 | Y | - | - |
| COL6A2 | Col6a2 | Y | - | - |
| COL4A2 | Col4a2 | Y | - | - |
| COL27A1 | Col27a1 | Y | - | - |
| CNKSR3 | Cnksr3 | Y | - | - |
| CNIH3 | Cnih3 | Y | - | - |
| CMAHP | Cmah | Y | - | - |
| CLIC6 | Clic6 | Y | - | - |
| CLCF1 | Clcf1 | Y | - | - |
| CKM | Ckm | Y | - | - |
| CISH | Cish | Y | - | - |
| CHMP4C | Chmp4c | Y | - | - |
| CERKL | Cerkl | Y | - | - |
| CEP135 | Cep135 | Y | - | - |
| CELSR2 | Celsr2 | Y | - | - |
| CELF6 | Celf6 | Y | - | - |
| CD6 | Cd6 | Y | - | - |
| CCR7 | Ccr7 | Y | - | - |
| CCDC40 | Ccdc40 | Y | - | - |
| CC2D2A | Cc2d2a | Y | - | - |
| CALR4P | Calr4 | Y | - | - |
| CALD1 | Cald1 | Y | - | - |
| CACNG2 | Cacng2 | Y | - | - |
| CACNB4 | Cacnb4 | Y | - | - |
| NA | C530044C16Rik | Y | - | - |
| KIAA1211 | C530008M17Rik | Y | - | - |
| C1QC | C1qc | Y | - | - |
| BTBD3 | Btbd3 | Y | - | - |
| BPTF | Bptf | Y | - | - |
| BOP1 | Bop1 | Y | - | - |
| BMP7 | Bmp7 | Y | - | - |
| BDNF | Bdnf | Y | - | - |
| BCAS3 | Bcas3 | Y | - | - |
| LPPR3 | BC005764 | Y | - | - |
| NA | BB014433 | Y | - | - |
| NA | B930018H19Rik | Y | - | - |
| B4GALNT3 | B4galnt3 | Y | - | - |
| AVEN | Aven | Y | - | - |
| AUTS2 | Auts2 | Y | - | - |
| NA | Asic1 | Y | - | - |
| ARMC2 | Armc2 | Y | - | - |
| ARHGEF40 | Arhgef40 | Y | - | - |
| ARHGEF38 | Arhgef38 | Y | - | - |
| ARHGAP9 | Arhgap9 | Y | - | - |
| ARHGAP23 | Arhgap23 | Y | - | - |
| APLP1 | Aplp1 | Y | - | - |
| ANTXR2 | Antxr2 | Y | - | - |
| ANKFN1 | Ankfn1 | Y | - | - |
| AMPD1 | Ampd1 | Y | - | - |
| AGAP1 | Agap1 | Y | - | - |
| ADORA2A | Adora2a | Y | - | - |
| ADORA1 | Adora1 | Y | - | - |
| ADARB1 | Adarb1 | Y | - | - |
| ADAM18 | Adam18 | Y | - | - |
| ADAM11 | Adam11 | Y | - | - |
| ACY1 | Acy1 | Y | - | - |
| ACVR2B | Acvr2b | Y | - | - |
| ACTL6B | Actl6b | Y | - | - |
| ABCC2 | Abcc2 | Y | - | - |
| ABCA4 | Abca4 | Y | - | - |
| NA | A630023A22Rik | Y | - | - |
| ASIP | a | Y | - | - |
| KIAA0889 | 9830001H06Rik | Y | - | - |
| NA | 5830418P13Rik | Y | - | - |
| LOC152586 | 4933434I20Rik | Y | - | - |
| NA | 4921506M07Rik | Y | - | - |
| NA | 2610307P16Rik | Y | - | - |
| NA | 1700085B03Rik | Y | - | - |
| C20orf85 | 1700021F07Rik | Y | - | - |
| 3-Sep | 3-Sep | Y | - | - |
| ZSCAN2 | Zscan2 | - | Y | - |
| ZMYND8 | Zmynd8 | - | Y | - |
| ZHX2 | Zhx2 | - | Y | - |
| ZNF710 | Zfp710 | - | Y | - |
| ZNF703 | Zfp703 | - | Y | - |
| ZNF76 | Zfp523 | - | Y | - |
| ZNF316 | Zfp316 | - | Y | - |
| ZNF205 | Zfp13 | - | Y | - |
| ZFHX3 | Zfhx3 | - | Y | - |
| ZBTB20 | Zbtb20 | - | Y | - |
| WSCD2 | Wscd2 | - | Y | - |
| WFDC1 | Wfdc1 | - | Y | - |
| NA | Wdr95 | - | Y | - |
| WDR65 | Wdr65 | - | Y | - |
| WDFY3 | Wdfy3 | - | Y | - |
| NA | Wbp1l | - | Y | - |
| NA | Vstm4 | - | Y | - |
| NA | Vps51 | - | Y | - |
| VAT1L | Vat1l | - | Y | - |
| UVRAG | Uvrag | - | Y | - |
| USP6NL | Usp6nl | - | Y | - |
| USP49 | Usp49 | - | Y | - |
| USH1G | Ush1g | - | Y | - |
| USH1C | Ush1c | - | Y | - |
| UNC5D | Unc5d | - | Y | - |
| UGGT1 | Uggt1 | - | Y | - |
| TXNDC5 | Txndc5 | - | Y | - |
| TWIST2 | Twist2 | - | Y | - |
| TTLL8 | Ttll8 | - | Y | - |
| TTLL6 | Ttll6 | - | Y | - |
| TTC29 | Ttc29 | - | Y | - |
| TTBK1 | Ttbk1 | - | Y | - |
| TSGA10 | Tsga10 | - | Y | - |
| TRUB1 | Trub1 | - | Y | - |
| TRIM9 | Trim9 | - | Y | - |
| TRIM47 | Trim47 | - | Y | - |
| TRIM37 | Trim37 | - | Y | - |
| TPSG1 | Tpsg1 | - | Y | - |
| TPH1 | Tph1 | - | Y | - |
| TOB2 | Tob2 | - | Y | - |
| TNNI3K | Tnni3k | - | Y | - |
| TNFRSF9 | Tnfrsf9 | - | Y | - |
| TMPRSS15 | Tmprss15 | - | Y | - |
| TMPRSS13 | Tmprss13 | - | Y | - |
| TMEM232 | Tmem232 | - | Y | - |
| TMEM198 | Tmem198 | - | Y | - |
| TMEM178 | Tmem178 | - | Y | - |
| TMEM150C | Tmem150c | - | Y | - |
| TMEM143 | Tmem143 | - | Y | - |
| TMEM132B | Tmem132b | - | Y | - |
| TMEM120B | Tmem120b | - | Y | - |
| TMEM104 | Tmem104 | - | Y | - |
| TMC6 | Tmc6 | - | Y | - |
| TLR3 | Tlr3 | - | Y | - |
| THEG | Theg | - | Y | - |
| THA1P | Tha1 | - | Y | - |
| TGFBR3 | Tgfbr3 | - | Y | - |
| TGFA | Tgfa | - | Y | - |
| TFAP2C | Tfap2c | - | Y | - |
| NA | Tenm2 | - | Y | - |
| TECTA | Tecta | - | Y | - |
| TEAD3 | Tead3 | - | Y | - |
| TCP11 | Tcp11 | - | Y | - |
| TBXA2R | Tbxa2r | - | Y | - |
| TBK1 | Tbk1 | - | Y | - |
| TBC1D17 | Tbc1d17 | - | Y | - |
| TAC3 | Tac2 | - | Y | - |
| SYT7 | Syt7 | - | Y | - |
| SYT13 | Syt13 | - | Y | - |
| SYT1 | Syt1 | - | Y | - |
| SYN3 | Syn3 | - | Y | - |
| SYDE2 | Syde2 | - | Y | - |
| SUSD4 | Susd4 | - | Y | - |
| SULT4A1 | Sult4a1 | - | Y | - |
| STOX1 | Stox1 | - | Y | - |
| ST8SIA5 | St8sia5 | - | Y | - |
| NA | Ssc5d | - | Y | - |
| NA | Sptb | - | Y | - |
| SPRED3 | Spred3 | - | Y | - |
| SPOCK1 | Spock1 | - | Y | - |
| SPARCL1 | Sparcl1 | - | Y | - |
| SOX7 | Sox7 | - | Y | - |
| SORBS2 | Sorbs2 | - | Y | - |
| SOD3 | Sod3 | - | Y | - |
| SNX11 | Snx11 | - | Y | - |
| SNRPN | Snrpn | - | Y | - |
| SMAD9 | Smad9 | - | Y | - |
| NA | Slco1a6 | - | Y | - |
| SLC8A1 | Slc8a1 | - | Y | - |
| SLC6A5 | Slc6a5 | - | Y | - |
| SLC39A4 | Slc39a4 | - | Y | - |
| SLC38A2 | Slc38a2 | - | Y | - |
| SLC2A9 | Slc2a9 | - | Y | - |
| SLC2A5 | Slc2a5 | - | Y | - |
| SLC25A21 | Slc25a21 | - | Y | - |
| SLC24A1 | Slc24a1 | - | Y | - |
| SLC1A3 | Slc1a3 | - | Y | - |
| SLC14A2 | Slc14a2 | - | Y | - |
| SLC13A3 | Slc13a3 | - | Y | - |
| SLC12A1 | Slc12a1 | - | Y | - |
| SIRT4 | Sirt4 | - | Y | - |
| SIK1 | Sik1 | - | Y | - |
| SH3BP4 | Sh3bp4 | - | Y | - |
| SH2D3C | Sh2d3c | - | Y | - |
| SGSM2 | Sgsm2 | - | Y | - |
| SF1 | Sf1 | - | Y | - |
| SEMA6C | Sema6c | - | Y | - |
| SEMA4F | Sema4f | - | Y | - |
| SDK1 | Sdk1 | - | Y | - |
| SCN8A | Scn8a | - | Y | - |
| SCN11A | Scn11a | - | Y | - |
| SCML4 | Scml4 | - | Y | - |
| SBK2 | Sbk2 | - | Y | - |
| SARM1 | Sarm1 | - | Y | - |
| SAMSN1 | Samsn1 | - | Y | - |
| SAMD14 | Samd14 | - | Y | - |
| RUNX3 | Runx3 | - | Y | - |
| RTN2 | Rtn2 | - | Y | - |
| RTN1 | Rtn1 | - | Y | - |
| RRP15 | Rrp15 | - | Y | - |
| RPS6KB2 | Rps6kb2 | - | Y | - |
| RP1L1 | Rp1l1 | - | Y | - |
| ROR1 | Ror1 | - | Y | - |
| ROPN1 | Ropn1 | - | Y | - |
| ROBO3 | Robo3 | - | Y | - |
| RIMKLA | Rimkla | - | Y | - |
| RIMBP2 | Rimbp2 | - | Y | - |
| RHPN1 | Rhpn1 | - | Y | - |
| RHOBTB2 | Rhobtb2 | - | Y | - |
| RFX8 | Rfx8 | - | Y | - |
| RBBP6 | Rbbp6 | - | Y | - |
| RASSF4 | Rassf4 | - | Y | - |
| RASSF3 | Rassf3 | - | Y | - |
| RASIP1 | Rasip1 | - | Y | - |
| RAPGEF3 | Rapgef3 | - | Y | - |
| RACGAP1 | Racgap1 | - | Y | - |
| RAB5C | Rab5c | - | Y | - |
| RAB3A | Rab3a | - | Y | - |
| QRFPR | Qrfpr | - | Y | - |
| PTPRB | Ptprb | - | Y | - |
| PTGIS | Ptgis | - | Y | - |
| PTCHD2 | Ptchd2 | - | Y | - |
| PSTPIP2 | Pstpip2 | - | Y | - |
| PSMG3 | Psmg3 | - | Y | - |
| PSME1 | Psme1 | - | Y | - |
| PRKD2 | Prkd2 | - | Y | - |
| PRKCE | Prkce | - | Y | - |
| PRIMA1 | Prima1 | - | Y | - |
| PPP2R2B | Ppp2r2b | - | Y | - |
| PPP1R9A | Ppp1r9a | - | Y | - |
| PPAP2B | Ppap2b | - | Y | - |
| POU6F2 | Pou6f2 | - | Y | - |
| POU6F1 | Pou6f1 | - | Y | - |
| PLXDC2 | Plxdc2 | - | Y | - |
| PLXDC1 | Plxdc1 | - | Y | - |
| PLEKHH3 | Plekhh3 | - | Y | - |
| PLCXD3 | Plcxd3 | - | Y | - |
| PLCB4 | Plcb4 | - | Y | - |
| PLCB1 | Plcb1 | - | Y | - |
| PLBD1 | Plbd1 | - | Y | - |
| PLA2G2C | Pla2g2c | - | Y | - |
| PLA2G1B | Pla2g1b | - | Y | - |
| PKLR | Pklr | - | Y | - |
| PIWIL2 | Piwil2 | - | Y | - |
| NA | Pinc | - | Y | - |
| PIK3C2G | Pik3c2g | - | Y | - |
| PIK3C2B | Pik3c2b | - | Y | - |
| PHYHIP | Phyhip | - | Y | - |
| PHKG1 | Phkg1 | - | Y | - |
| PHF17 | Phf17 | - | Y | - |
| PHC2 | Phc2 | - | Y | - |
| PHACTR4 | Phactr4 | - | Y | - |
| PDZRN4 | Pdzrn4 | - | Y | - |
| PDE7A | Pde7a | - | Y | - |
| PDE4B | Pde4b | - | Y | - |
| PDE11A | Pde11a | - | Y | - |
| PDCL3 | Pdcl3 | - | Y | - |
| PDCD11 | Pdcd11 | - | Y | - |
| PCSK6 | Pcsk6 | - | Y | - |
| PCMTD1 | Pcmtd1 | - | Y | - |
| PCIF1 | Pcif1 | - | Y | - |
| PCDHGB6 | Pcdhgb6 | - | Y | - |
| PCDHGA9 | Pcdhga9 | - | Y | - |
| PCDHAC1 | Pcdhac1 | - | Y | - |
| PCDHA3 | Pcdha3 | - | Y | - |
| PCDHA2 | Pcdha2 | - | Y | - |
| PCDHA1 | Pcdha1 | - | Y | - |
| PAX5 | Pax5 | - | Y | - |
| PARK2 | Park2 | - | Y | - |
| PAQR3 | Paqr3 | - | Y | - |
| PAPLN | Papln | - | Y | - |
| PAH | Pah | - | Y | - |
| PACRG | Pacrg | - | Y | - |
| P2RX7 | P2rx7 | - | Y | - |
| OTOP2 | Otop2 | - | Y | - |
| OSBPL3 | Osbpl3 | - | Y | - |
| OPRD1 | Oprd1 | - | Y | - |
| NA | Olfr283 | - | Y | - |
| OLFM2 | Olfm2 | - | Y | - |
| NUAK1 | Nuak1 | - | Y | - |
| NRXN1 | Nrxn1 | - | Y | - |
| NRG4 | Nrg4 | - | Y | - |
| NRAP | Nrap | - | Y | - |
| NR2F6 | Nr2f6 | - | Y | - |
| NR1H3 | Nr1h3 | - | Y | - |
| NPR2 | Npr2 | - | Y | - |
| NPAS1 | Npas1 | - | Y | - |
| NOD1 | Nod1 | - | Y | - |
| NFKBIE | Nfkbie | - | Y | - |
| NFATC4 | Nfatc4 | - | Y | - |
| NEURL | Neurl1a | - | Y | - |
| GNAS-AS1 | Nespas | - | Y | - |
| NEK7 | Nek7 | - | Y | - |
| NCOR1 | Ncor1 | - | Y | - |
| NCF1C | Ncf1 | - | Y | - |
| NCAM1 | Ncam1 | - | Y | - |
| NCALD | Ncald | - | Y | - |
| NAV3 | Nav3 | - | Y | - |
| NAV1 | Nav1 | - | Y | - |
| NA | Myrfl | - | Y | - |
| MYOZ1 | Myoz1 | - | Y | - |
| MYO5C | Myo5c | - | Y | - |
| MYO18B | Myo18b | - | Y | - |
| MYO10 | Myo10 | - | Y | - |
| MYCL1 | Mycl1 | - | Y | - |
| MTHFD1L | Mthfd1l | - | Y | - |
| MSI2 | Msi2 | - | Y | - |
| MPO | Mpo | - | Y | - |
| MPL | Mpl | - | Y | - |
| MOB3B | Mob3b | - | Y | - |
| MMP9 | Mmp9 | - | Y | - |
| MMD | Mmd | - | Y | - |
| MLPH | Mlph | - | Y | - |
| MLLT3 | Mllt3 | - | Y | - |
| NA | Mir466n | - | Y | - |
| NA | Mir1a-1 | - | Y | - |
| MGST3 | Mgst3 | - | Y | - |
| MGLL | Mgll | - | Y | - |
| MEX3A | Mex3a | - | Y | - |
| NA | Mettl24 | - | Y | - |
| MEGF11 | Megf11 | - | Y | - |
| MEGF10 | Megf10 | - | Y | - |
| MDGA1 | Mdga1 | - | Y | - |
| NA | Mb21d2 | - | Y | - |
| MAST1 | Mast1 | - | Y | - |
| MAPK4 | Mapk4 | - | Y | - |
| MAP3K6 | Map3k6 | - | Y | - |
| MAP3K1 | Map3k1 | - | Y | - |
| NA | Map1b | - | Y | - |
| MAMDC2 | Mamdc2 | - | Y | - |
| MAGI1 | Magi1 | - | Y | - |
| NA | Lurap1 | - | Y | - |
| LRRC4B | Lrrc4b | - | Y | - |
| LRRC24 | Lrrc24 | - | Y | - |
| LRRC1 | Lrrc1 | - | Y | - |
| LRMP | Lrmp | - | Y | - |
| LRIT3 | Lrit3 | - | Y | - |
| LRFN4 | Lrfn4 | - | Y | - |
| LRFN3 | Lrfn3 | - | Y | - |
| LRFN2 | Lrfn2 | - | Y | - |
| LOXL3 | Loxl3 | - | Y | - |
| LMTK3 | Lmtk3 | - | Y | - |
| LMOD1 | Lmod1 | - | Y | - |
| LMNB1 | Lmnb1 | - | Y | - |
| LINGO3 | Lingo3 | - | Y | - |
| LIMA1 | Lima1 | - | Y | - |
| LHX9 | Lhx9 | - | Y | - |
| LASP1 | Lasp1 | - | Y | - |
| LAMA2 | Lama2 | - | Y | - |
| LACE1 | Lace1 | - | Y | - |
| KPNA6 | Kpna6 | - | Y | - |
| KLF13 | Klf13 | - | Y | - |
| KISS1R | Kiss1r | - | Y | - |
| KIF26A | Kif26a | - | Y | - |
| KIF19 | Kif19a | - | Y | - |
| KHSRP | Khsrp | - | Y | - |
| KCP | Kcp | - | Y | - |
| KCNN4 | Kcnn4 | - | Y | - |
| KCNK6 | Kcnk6 | - | Y | - |
| KCNK4 | Kcnk4 | - | Y | - |
| KCNK12 | Kcnk12 | - | Y | - |
| KCNJ6 | Kcnj6 | - | Y | - |
| KCNIP3 | Kcnip3 | - | Y | - |
| KCNIP1 | Kcnip1 | - | Y | - |
| KCNAB1 | Kcnab1 | - | Y | - |
| KCNA10 | Kcna10 | - | Y | - |
| JPH2 | Jph2 | - | Y | - |
| JAKMIP2 | Jakmip2 | - | Y | - |
| ITPKB | Itpkb | - | Y | - |
| ITK | Itk | - | Y | - |
| ITGA9 | Itga9 | - | Y | - |
| ITGA2 | Itga2 | - | Y | - |
| IRF8 | Irf8 | - | Y | - |
| IQSEC3 | Iqsec3 | - | Y | - |
| IL2RA | Il2ra | - | Y | - |
| IGFBPL1 | Igfbpl1 | - | Y | - |
| IGF2R | Igf2r | - | Y | - |
| IGDCC4 | Igdcc4 | - | Y | - |
| HSPA12A | Hspa12a | - | Y | - |
| HS6ST3 | Hs6st3 | - | Y | - |
| HS3ST5 | Hs3st5 | - | Y | - |
| HPCA | Hpca | - | Y | - |
| HOXA3 | Hoxa3 | - | Y | - |
| HLF | Hlf | - | Y | - |
| HK1 | Hk1 | - | Y | - |
| HEATR8 | Heatr8 | - | Y | - |
| HCN4 | Hcn4 | - | Y | - |
| HAP1 | Hap1 | - | Y | - |
| GSTO2 | Gsto2 | - | Y | - |
| GRM8 | Grm8 | - | Y | - |
| GRID1 | Grid1 | - | Y | - |
| GRIA1 | Gria1 | - | Y | - |
| GRB7 | Grb7 | - | Y | - |
| GRAP2 | Grap2 | - | Y | - |
| GPR133 | Gpr133 | - | Y | - |
| GPR123 | Gpr123 | - | Y | - |
| GPD1 | Gpd1 | - | Y | - |
| GNAO1 | Gnao1 | - | Y | - |
| GNAI1 | Gnai1 | - | Y | - |
| NA | Gm9899 | - | Y | - |
| NA | Gm872 | - | Y | - |
| NA | Gm6307 | - | Y | - |
| NA | Gm6249 | - | Y | - |
| NA | Gm5424 | - | Y | - |
| NA | Gm53 | - | Y | - |
| NA | Gm16063 | - | Y | - |
| NA | Gm1110 | - | Y | - |
| NA | Gm10863 | - | Y | - |
| GIPC3 | Gipc3 | - | Y | - |
| GFRA1 | Gfra1 | - | Y | - |
| GFOD1 | Gfod1 | - | Y | - |
| GDF10 | Gdf10 | - | Y | - |
| GALNT14 | Galnt14 | - | Y | - |
| GAD2 | Gad2 | - | Y | - |
| FZD5 | Fzd5 | - | Y | - |
| FZD1 | Fzd1 | - | Y | - |
| FUZ | Fuz | - | Y | - |
| FSTL4 | Fstl4 | - | Y | - |
| FOXP4 | Foxp4 | - | Y | - |
| FNTB | Fntb | - | Y | - |
| FNDC7 | Fndc7 | - | Y | - |
| FLT4 | Flt4 | - | Y | - |
| FHL2 | Fhl2 | - | Y | - |
| FHAD1 | Fhad1 | - | Y | - |
| FGF17 | Fgf17 | - | Y | - |
| FBLN7 | Fbln7 | - | Y | - |
| FAT1 | Fat1 | - | Y | - |
| FARSB | Farsb | - | Y | - |
| FAM57B | Fam57b | - | Y | - |
| FAM46B | Fam46b | - | Y | - |
| NA | Fam219a | - | Y | - |
| NA | Fam211a | - | Y | - |
| FAM19A2 | Fam19a2 | - | Y | - |
| FAM129C | Fam129c | - | Y | - |
| FAM129A | Fam129a | - | Y | - |
| EYA2 | Eya2 | - | Y | - |
| EXTL1 | Extl1 | - | Y | - |
| ERC2 | Erc2 | - | Y | - |
| EPS8L2 | Eps8l2 | - | Y | - |
| EPHX2 | Ephx2 | - | Y | - |
| EPHB1 | Ephb1 | - | Y | - |
| EMP2 | Emp2 | - | Y | - |
| ELK3 | Elk3 | - | Y | - |
| ELFN2 | Elfn2 | - | Y | - |
| EHMT2 | Ehmt2 | - | Y | - |
| EGR3 | Egr3 | - | Y | - |
| EFNA5 | Efna5 | - | Y | - |
| EFCAB3 | Efcab3 | - | Y | - |
| EDARADD | Edaradd | - | Y | - |
| ECHDC3 | Echdc3 | - | Y | - |
| NA | E130006D01Rik | - | Y | - |
| DYX1C1 | Dyx1c1 | - | Y | - |
| DYRK1B | Dyrk1b | - | Y | - |
| DUSP8 | Dusp8 | - | Y | - |
| DUSP15 | Dusp15 | - | Y | - |
| DUOX2 | Duox2 | - | Y | - |
| DTNA | Dtna | - | Y | - |
| DPPA3 | Dppa3 | - | Y | - |
| DOC2A | Doc2a | - | Y | - |
| DNAJC6 | Dnajc6 | - | Y | - |
| DNAJB13 | Dnajb13 | - | Y | - |
| DNAI2 | Dnaic2 | - | Y | - |
| DMRT1 | Dmrt1 | - | Y | - |
| DLK1 | Dlk1 | - | Y | - |
| DLG5 | Dlg5 | - | Y | - |
| DLG4 | Dlg4 | - | Y | - |
| DENND4A | Dennd4a | - | Y | - |
| DEF6 | Def6 | - | Y | - |
| DEDD2 | Dedd2 | - | Y | - |
| DDAH1 | Ddah1 | - | Y | - |
| DCTN6 | Dctn6 | - | Y | - |
| DCLRE1B | Dclre1b | - | Y | - |
| DCDC2 | Dcdc2a | - | Y | - |
| DCC | Dcc | - | Y | - |
| DAPP1 | Dapp1 | - | Y | - |
| DACT2 | Dact2 | - | Y | - |
| C12orf56 | D930020B18Rik | - | Y | - |
| KIAA1755 | D630003M21Rik | - | Y | - |
| NA | D330022K07Rik | - | Y | - |
| NA | Cyp4f41-ps | - | Y | - |
| CYP4F8 | Cyp4f17 | - | Y | - |
| CYP1A1 | Cyp1a1 | - | Y | - |
| CXXC5 | Cxxc5 | - | Y | - |
| CXCR5 | Cxcr5 | - | Y | - |
| CUEDC1 | Cuedc1 | - | Y | - |
| CTH | Cth | - | Y | - |
| CTCFL | Ctcfl | - | Y | - |
| NA | Cstad | - | Y | - |
| CRYBB3 | Crybb3 | - | Y | - |
| CPNE7 | Cpne7 | - | Y | - |
| CPLX2 | Cplx2 | - | Y | - |
| COMMD1 | Commd1 | - | Y | - |
| COLEC11 | Colec11 | - | Y | - |
| COL6A6 | Col6a6 | - | Y | - |
| COL5A3 | Col5a3 | - | Y | - |
| COL4A4 | Col4a4 | - | Y | - |
| CNTNAP1 | Cntnap1 | - | Y | - |
| CNTN5 | Cntn5 | - | Y | - |
| CNTFR | Cntfr | - | Y | - |
| CNGB1 | Cngb1 | - | Y | - |
| NA | Cmip | - | Y | - |
| CLSTN2 | Clstn2 | - | Y | - |
| TMEM114 | Cldn26 | - | Y | - |
| CLDN14 | Cldn14 | - | Y | - |
| CHST8 | Chst8 | - | Y | - |
| CHST5 | Chst5 | - | Y | - |
| CHST4 | Chst4 | - | Y | - |
| CHL1 | Chl1 | - | Y | - |
| CELF5 | Celf5 | - | Y | - |
| CDKL3 | Cdkl3 | - | Y | - |
| CDK19 | Cdk19 | - | Y | - |
| CDHR3 | Cdhr3 | - | Y | - |
| CDH3 | Cdh3 | - | Y | - |
| CDH20 | Cdh20 | - | Y | - |
| CDH15 | Cdh15 | - | Y | - |
| CDH13 | Cdh13 | - | Y | - |
| CDH10 | Cdh10 | - | Y | - |
| CD9 | Cd9 | - | Y | - |
| CD37 | Cd37 | - | Y | - |
| CD22 | Cd22 | - | Y | - |
| CCNJL | Ccnjl | - | Y | - |
| CCDC42B | Ccdc42b | - | Y | - |
| CCDC30 | Ccdc30 | - | Y | - |
| CCDC155 | Ccdc155 | - | Y | - |
| CCDC113 | Ccdc113 | - | Y | - |
| CCDC111 | Ccdc111 | - | Y | - |
| CAPN3 | Capn3 | - | Y | - |
| NA | Camkmt | - | Y | - |
| CALN1 | Caln1 | - | Y | - |
| CALCRL | Calcrl | - | Y | - |
| CALCR | Calcr | - | Y | - |
| CACNA1S | Cacna1s | - | Y | - |
| CACNA1H | Cacna1h | - | Y | - |
| CACNA1B | Cacna1b | - | Y | - |
| C1QTNF2 | C1qtnf2 | - | Y | - |
| BTRC | Btrc | - | Y | - |
| BTNL9 | Btnl9 | - | Y | - |
| BPIFB1 | Bpifb1 | - | Y | - |
| BMPER | Bmper | - | Y | - |
| BMP3 | Bmp3 | - | Y | - |
| BID | Bid | - | Y | - |
| BICC1 | Bicc1 | - | Y | - |
| BEST3 | Best3 | - | Y | - |
| BCMO1 | Bcmo1 | - | Y | - |
| BCL3 | Bcl3 | - | Y | - |
| BCAT1 | Bcat1 | - | Y | - |
| BCAS1 | Bcas1 | - | Y | - |
| LOC732206 | BC100451 | - | Y | - |
| C7orf43 | BC037034 | - | Y | - |
| BATF3 | Batf3 | - | Y | - |
| BASP1 | Basp1 | - | Y | - |
| B4GALT2 | B4galt2 | - | Y | - |
| NA | B430306N03Rik | - | Y | - |
| ATP1A3 | Atp1a3 | - | Y | - |
| ATP11A | Atp11a | - | Y | - |
| ATG7 | Atg7 | - | Y | - |
| ASPG | Aspg | - | Y | - |
| NA | Asic2 | - | Y | - |
| ASB18 | Asb18 | - | Y | - |
| ART3 | Art3 | - | Y | - |
| ARSB | Arsb | - | Y | - |
| ARPP21 | Arpp21 | - | Y | - |
| NA | Armc12 | - | Y | - |
| ARL10 | Arl10 | - | Y | - |
| ARID3C | Arid3c | - | Y | - |
| ARID3A | Arid3a | - | Y | - |
| ARID1B | Arid1b | - | Y | - |
| ARHGEF4 | Arhgef4 | - | Y | - |
| NA | Arhgef28 | - | Y | - |
| ARHGAP40 | Arhgap40 | - | Y | - |
| ARHGAP28 | Arhgap28 | - | Y | - |
| ARHGAP24 | Arhgap24 | - | Y | - |
| APBA2 | Apba2 | - | Y | - |
| AOAH | Aoah | - | Y | - |
| ANP32A | Anp32a | - | Y | - |
| ANO4 | Ano4 | - | Y | - |
| ANO1 | Ano1 | - | Y | - |
| ANKRD6 | Ankrd6 | - | Y | - |
| ANKRD34B | Ankrd34b | - | Y | - |
| ANKRD13A | Ankrd13a | - | Y | - |
| AMOTL1 | Amotl1 | - | Y | - |
| ALX3 | Alx3 | - | Y | - |
| ALDH3A1 | Aldh3a1 | - | Y | - |
| NA | Akp3 | - | Y | - |
| AKAP6 | Akap6 | - | Y | - |
| NA | AI854517 | - | Y | - |
| C6orf132 | AI661453 | - | Y | - |
| AFF3 | Aff3 | - | Y | - |
| ADRBK2 | Adrbk2 | - | Y | - |
| ADRBK1 | Adrbk1 | - | Y | - |
| ADCYAP1R1 | Adcyap1r1 | - | Y | - |
| ADCY2 | Adcy2 | - | Y | - |
| ADCY1 | Adcy1 | - | Y | - |
| ADAMTS4 | Adamts4 | - | Y | - |
| ADAMTS2 | Adamts2 | - | Y | - |
| ADA | Ada | - | Y | - |
| ACTN3 | Actn3 | - | Y | - |
| ACTN1 | Actn1 | - | Y | - |
| ACE | Ace | - | Y | - |
| ACAN | Acan | - | Y | - |
| ABCG8 | Abcg8 | - | Y | - |
| ABCC8 | Abcc8 | - | Y | - |
| ABCC3 | Abcc3 | - | Y | - |
| ABCC12 | Abcc12 | - | Y | - |
| AARS2 | Aars2 | - | Y | - |
| NA | A430093F15Rik | - | Y | - |
| NA | 9130019P16Rik | - | Y | - |
| NA | 6430531B16Rik | - | Y | - |
| NA | 5830403M04Rik | - | Y | - |
| NA | 4930500F04Rik | - | Y | - |
| C22orf46 | 4930407I10Rik | - | Y | - |
| C2orf76 | 3110009E18Rik | - | Y | - |
| LOC100499467 | 2610035D17Rik | - | Y | - |
| NA | 2410003L11Rik | - | Y | - |
| NA | 2310065F04Rik | - | Y | - |
| NA | 2210039B01Rik | - | Y | - |
| KIAA1644 | 1810041L15Rik | - | Y | - |
| LOC643406 | 1700026D11Rik | - | Y | - |
| C10orf53 | 1700024G13Rik | - | Y | - |
| NA | 1700023F06Rik | - | Y | - |
| C4orf22 | 1700007G11Rik | - | Y | - |
| NA | 1300017J02Rik | - | Y | - |
| KIAA0317 | 1110018G07Rik | - | Y | - |
| 10-Mar | 10-Mar | - | Y | - |
| 1-Mar | 1-Mar | - | Y | - |
| ZNF664-FAM101A | NA | - | - | Y |
| ZNF503-AS1 | NA | - | - | Y |
| ZDHHC8P1 | NA | - | - | Y |
| VPS51 | NA | - | - | Y |
| UVSSA | NA | - | - | Y |
| USP6 | NA | - | - | Y |
| UNC80 | NA | - | - | Y |
| TTC28-AS1 | NA | - | - | Y |
| TSNARE1 | NA | - | - | Y |
| TOR4A | NA | - | - | Y |
| TMEM105 | NA | - | - | Y |
| TEX34 | NA | - | - | Y |
| TEN1-CDK3 | NA | - | - | Y |
| SLC22A31 | NA | - | - | Y |
| SEPT5-GP1BB | NA | - | - | Y |
| RUSC1-AS1 | NA | - | - | Y |
| RTEL1-TNFRSF6B | NA | - | - | Y |
| RPLP0P2 | NA | - | - | Y |
| RNF157-AS1 | NA | - | - | Y |
| RAX2 | NA | - | - | Y |
| RABL6 | NA | - | - | Y |
| PRR5-ARHGAP8 | NA | - | - | Y |
| POM121L9P | NA | - | - | Y |
| PLA2G4B | NA | - | - | Y |
| P2RX5-TAX1BP3 | NA | - | - | Y |
| NRON | NA | - | - | Y |
| NLRP7 | NA | - | - | Y |
| MIRLET7BHG | NA | - | - | Y |
| MIR99B | NA | - | - | Y |
| MIR657 | NA | - | - | Y |
| MIR4758 | NA | - | - | Y |
| MIR4667 | NA | - | - | Y |
| MIR4641 | NA | - | - | Y |
| MIR4489 | NA | - | - | Y |
| MIR4309 | NA | - | - | Y |
| MIR4292 | NA | - | - | Y |
| MIR3677 | NA | - | - | Y |
| MIR338 | NA | - | - | Y |
| MIR3065 | NA | - | - | Y |
| MIR200B | NA | - | - | Y |
| MIR1226 | NA | - | - | Y |
| MAPT-AS1 | NA | - | - | Y |
| MAP3K14-AS1 | NA | - | - | Y |
| MAFG-AS1 | NA | - | - | Y |
| LOC728752 | NA | - | - | Y |
| LOC728743 | NA | - | - | Y |
| LOC728613 | NA | - | - | Y |
| LOC642852 | NA | - | - | Y |
| LOC285692 | NA | - | - | Y |
| LOC285626 | NA | - | - | Y |
| LOC284751 | NA | - | - | Y |
| LOC284632 | NA | - | - | Y |
| LOC254099 | NA | - | - | Y |
| LOC219731 | NA | - | - | Y |
| LOC200772 | NA | - | - | Y |
| LOC149950 | NA | - | - | Y |
| LOC100996291 | NA | - | - | Y |
| LOC100508120 | NA | - | - | Y |
| LOC100507373 | NA | - | - | Y |
| LOC100131691 | NA | - | - | Y |
| LOC100130872 | NA | - | - | Y |
| LOC100130855 | NA | - | - | Y |
| LOC100128675 | NA | - | - | Y |
| LOC100128568 | NA | - | - | Y |
| LOC100128076 | NA | - | - | Y |
| LINC00669 | NA | - | - | Y |
| LINC00661 | NA | - | - | Y |
| LINC00523 | NA | - | - | Y |
| LINC00299 | NA | - | - | Y |
| KIAA1671 | NA | - | - | Y |
| IL26 | NA | - | - | Y |
| HELZ2 | NA | - | - | Y |
| HCG17 | NA | - | - | Y |
| HAR1B | NA | - | - | Y |
| GUSBP11 | NA | - | - | Y |
| GUCY2EP | NA | - | - | Y |
| GSE1 | NA | - | - | Y |
| GPR144 | NA | - | - | Y |
| FAM86A | NA | - | - | Y |
| FAM22G | NA | - | - | Y |
| EXD3 | NA | - | - | Y |
| EVPLL | NA | - | - | Y |
| ETV7 | NA | - | - | Y |
| ELMSAN1 | NA | - | - | Y |
| DGCR6L | NA | - | - | Y |
| DGCR5 | NA | - | - | Y |
| CROCCP3 | NA | - | - | Y |
| CATSPERD | NA | - | - | Y |
| C3P1 | NA | - | - | Y |
| C2orf48 | NA | - | - | Y |
| C1orf186 | NA | - | - | Y |
| C19orf26 | NA | - | - | Y |
| C17orf76-AS1 | NA | - | - | Y |
| C16orf11 | NA | - | - | Y |
| C10orf91 | NA | - | - | Y |
| AZU1 | NA | - | - | Y |
| APOBEC3A_B | NA | - | - | Y |
| AFAP1-AS1 | NA | - | - | Y |
| AATK-AS1 | NA | - | - | Y |
