# Supplementary material for: AICDA drives epigenetic heterogeneity and accelerates germinal center-derived lymphomagenesis
Source: Nat Commun. 2018 Jan 15;9:222. doi: 10.1038/s41467-017-02595-w (PMC5768781; doi:10.1038/s41467-017-02595-w)
Supplement: Supplementary file 5 — Supplementary Data 2 [file 41467_2017_2595_MOESM5_ESM.docx]

**Supplementary Data 2. Leading edge genes showing AICDA-perturbed, down-regulated genes in mouse and human lymphoma**

| VavP-Bcl2 Leading Edge Genes | Human DLBCL Leading Edge Genes |
| --- | --- |
| **Agap3** | **AGAP3** |
| **Bcl2** | **BCL2** |
| **Ccdc88c** | **CCDC88C** |
| **Fhod3** | **FHOD3** |
| **Fmnl1** | **FMNL1** |
| **Prkca** | **PRKCA** |
| **Rgs3** | **RGS3** |
| **Sema4d** | **SEMA4D** |
| Sept11 | ACAP3 |
| Abca7 | ACTN4 |
| Acy1 | ALDH1A3 |
| Agap2 | ALDH1L1 |
| Antxr2 | ANO9 |
| Anxa6 | APP |
| Arhgap9 | ARHGEF10L |
| Cacna1e | ARHGEF18 |
| Cacna1i | ARHGEF7 |
| Calhm2 | ARRB1 |
| Casc3 | ARSA |
| Ccr7 | ATXN1 |
| Cdt1 | AUTS2 |
| Cmah | AZI1 |
| Coro7 | BCR |
| Ctdp1 | BIN1 |
| Cul9 | C15orf39 |
| Dapp1 | C19orf38 |
| Ddx6 | C2 |
| Dgka | C3 |
| Dgkz | CABLES1 |
| Dnm3 | CACNA2D4 |
| Dst | CAMK2B |
| Ebf1 | CARD11 |
| Evl | CBFA2T3 |
| Fbxl12 | CCDC102A |
| Fnbp1 | CCND1 |
| Fto | CDC42BPB |
| Gaa | CDC42BPG |
| Glcci1 | CDC42EP2 |
| Igsf3 | CDC42EP4 |
| Il6ra | CDH23 |
| Inadl | CELF2 |
| Irf4 | CELSR3 |
| Ivd | CERS4 |
| Jdp2 | CHST11 |
| Gse1 | CHST15 |
| Etl4 | CHTF18 |
| Lrrc33 | CLCN7 |
| Map3k14 | CLEC16A |
| Map4k4 | CLIP2 |
| Mfhas1 | CLTB |
| Mll2 | CMIP |
| Myo18a | CNST |
| Myof | COL16A1 |
| Cmip | COL5A1 |
| Nfatc2 | COL6A3 |
| Nfkbie | COL9A3 |
| Notch1 | COMP |
| Nrp2 | CPNE5 |
| Pbx1 | CPNE7 |
| Pdcd11 | CPXM2 |
| Prkce | CREB5 |
| Prrc2b | CTSD |
| Ptpdc1 | CTSW |
| Ptpn13 | CTTN |
| Ptpre | CYFIP1 |
| R3hdm2 | DEF8 |
| Rab11fip4 | DFFB |
| Ranbp10 | DFNB31 |
| Rell1 | DHX58 |
| Rnf123 | DNAH1 |
| Rnf144a | DNAJC11 |
| Rnf169 | DOCK6 |
| Satb1 | DOK3 |
| Sdc4 | DTNB |
| Sergef | DVL1 |
| Setd1b | EEF1A2 |
| Slc36a1 | EEF2K |
| Slc43a2 | FAM129B |
| Slc4a8 | FAM134C |
| Snx30 | FAM59A |
| Ssh1 | FBLN1 |
| Stk10 | FBN1 |
| Tcirg1 | FCHO1 |
| Trf | FKBP10 |
| Tln1 | FLNB |
| Tmcc3 | FN1 |
| Tmco7 | FOXN3 |
| Tnrc6b | FSTL5 |
| Tox2 | GALNT6 |
| Trappc9 | GAS6 |
| Txndc5 | GDPD5 |
| Unc119b | GFPT2 |
| Usp48 | GLI2 |
| Wdr91 | GMDS |
| Wwp2 | GRAMD4 |
| Xylt1 | GRK5 |
| Zfp362 | H6PD |
|  | HR |
|  | HSPG2 |
|  | ICOSLG |
|  | IGFBP2 |
|  | IGHMBP2 |
|  | IKBKB |
|  | IL12RB1 |
|  | IL2RB |
|  | IQCE |
|  | IRF7 |
|  | ITGAX |
|  | ITGB2 |
|  | ITGB5 |
|  | ITPR2 |
|  | KANK2 |
|  | KCNAB2 |
|  | KIFC3 |
|  | KLHDC4 |
|  | KLHL29 |
|  | LHPP |
|  | LMF1 |
|  | LMNA |
|  | LOC728743 |
|  | LPCAT1 |
|  | LRP1 |
|  | LSP1 |
|  | LTBP2 |
|  | LTBP3 |
|  | MAN2B2 |
|  | MATK |
|  | MEGF6 |
|  | MGRN1 |
|  | MIB2 |
|  | MICAL1 |
|  | MTA3 |
|  | MUM1 |
|  | MXD4 |
|  | MYH9 |
|  | MYO15B |
|  | MYO7B |
|  | NAT6 |
|  | NCOR2 |
|  | NCS1 |
|  | NEK6 |
|  | NFATC1 |
|  | NLRP4 |
|  | NOS3 |
|  | NOTCH3 |
|  | NPEPPS |
|  | OBSL1 |
|  | P2RX5 |
|  | PADI2 |
|  | PAPD7 |
|  | PDGFRB |
|  | PDLIM7 |
|  | PIEZO1 |
|  | PIK3R5 |
|  | PIP5K1C |
|  | PITPNC1 |
|  | PLA2G4C |
|  | PLEKHM1 |
|  | PLXNA1 |
|  | PLXND1 |
|  | PREX1 |
|  | PSAP |
|  | PTK2 |
|  | PTPRJ |
|  | PTPRS |
|  | PVRL1 |
|  | RAB11FIP3 |
|  | RAB40C |
|  | RALB |
|  | RASA3 |
|  | RASGRP2 |
|  | RECQL4 |
|  | RELT |
|  | RIBC2 |
|  | RIN2 |
|  | RNF130 |
|  | RNF166 |
|  | RXRA |
|  | SBF1 |
|  | SCARB1 |
|  | SERINC5 |
|  | SERPINB6 |
|  | SERPINF1 |
|  | SFMBT2 |
|  | SHANK3 |
|  | SLC12A4 |
|  | SLC23A2 |
|  | SLC35F2 |
|  | SLC7A5 |
|  | SLC9A3R2 |
|  | SMAD3 |
|  | SMPD3 |
|  | SORL1 |
|  | SPNS2 |
|  | SPTBN5 |
|  | SRC |
|  | SREBF1 |
|  | SREBF2 |
|  | SSH2 |
|  | STAB1 |
|  | STAT5A |
|  | SUOX |
|  | SYDE1 |
|  | TAGLN |
|  | TBC1D8 |
|  | TCF3 |
|  | TESK1 |
|  | TGM2 |
|  | TIAM2 |
|  | TIE1 |
|  | TLL2 |
|  | TMEM129 |
|  | TNFAIP2 |
|  | TNFRSF1B |
|  | TNFRSF25 |
|  | TNFRSF4 |
|  | TNK2 |
|  | TNS1 |
|  | TPCN2 |
|  | TRIM8 |
|  | TTYH3 |
|  | UBASH3B |
|  | UTRN |
|  | VAV2 |
|  | VCL |
|  | WDR90 |
|  | WDTC1 |
|  | WISP2 |
|  | ZBTB48 |
|  | ZHX2 |
|  | ZNF423 |
|  | ZNF516 |
